# Genetic targeting of sprouting angiogenesis using Apln-CreER

**DOI:** 10.1038/ncomms7020

**Published:** 2015-01-19

**Authors:** Qiaozhen Liu, Tianyuan Hu, Lingjuan He, Xiuzhen Huang, Xueying Tian, Hui Zhang, Liang He, Wenjuan Pu, Libo Zhang, Heng Sun, Jing Fang, Ying Yu, Shengzhong Duan, Chaobo Hu, Lijian Hui, Haibin Zhang, Thomas Quertermous, Qingbo Xu, Kristy Red-Horse, Joshua D. Wythe, Bin Zhou

**Affiliations:** 1 Key Laboratory of Nutrition and Metabolism, Institute for Nutritional Sciences, Shanghai Institutes for Biological Sciences, Graduate School of the Chinese Academy of Sciences, Chinese Academy of Sciences, Yueyang Road 320, Shanghai 200031, China; 2 Life and Brain Center, Institute of Physiology I, University of Bonn, Sigmund-Freud-Strasse 25, 53105 Bonn, Germany; 3 Laboratory of Molecular Cell Biology, Institute of Biochemistry and Cell Biology, Shanghai Institutes for Biological Sciences, Chinese Academy of Sciences, Shanghai 200031, China; 4 Fifth Department of Hepatic Surgery, Eastern Hepatobilliary Surgery Hospital, Second Military Medical University, Shanghai 200433, China; 5 Department of Medicine, Stanford University School of Medicine, Stanford, California 94305, USA; 6 Cardiovascular Division, King’s College London British Heart Foundation Centre, London SE5 9NU, UK; 7 Department of Molecular Physiology and Biophysics, Cardiovascular Research Institute, Baylor College of Medicine, Houston, Texas 77030, USA; 8 CAS Center for Excellence in Brain Science, Shanghai Institutes for Biological Sciences, Chinese Academy of Sciences, Shanghai 200031, China

## Abstract

Under pathophysiological conditions in adults, endothelial cells (ECs) sprout from pre-existing blood vessels to form new ones by a process termed angiogenesis. During embryonic development, Apelin (APLN) is robustly expressed in vascular ECs. In adult mice, however, APLN expression in the vasculature is significantly reduced. Here we show that APLN expression is reactivated in adult ECs after ischaemia insults. In models of both injury ischaemia and tumor angiogenesis, we find that Apln-CreER genetically labels sprouting but not quiescent vasculature. By leveraging this specific activity, we demonstrate that abolishment of the VEGF–VEGFR2 signalling pathway as well as ablation of sprouting ECs diminished tumour vascularization and growth without compromising vascular homeostasis in other organs. Collectively, we show that Apln-CreER distinguishes sprouting vessels from stabilized vessels in multiple pathological settings. The Apln-CreER line described here will greatly aid future mechanistic studies in both vascular developmental biology and adult vascular diseases.

## Abstract (web summary)

Apelin expression is robust in embryonic but not in adult endothelial cells (ECs), where it can be reactivated by hypoxia. Liu *et al*. show that apelin-driven expression of Cre recombinase in mice can be used for labelling of, or gene ablation in, sprouting but not quiescent ECs in pathologies characterized by hypoxia.

## Graphical abstract

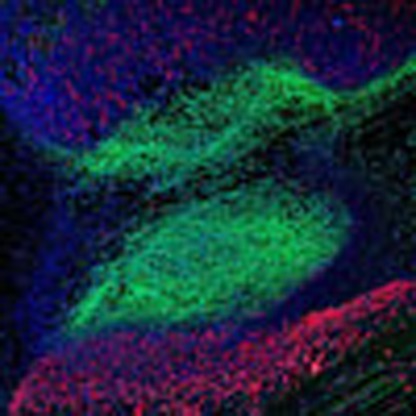

Vascular diseases such as coronary artery diseases and stroke are the leading cause of death worldwide. In these pathological conditions, hypoxia in ischaemic tissues stimulates new blood vessels to sprout from pre-existing vessels^1^,^2^. Therefore, after injury or insult, a subset of blood vessel endothelial cells (ECs) becomes activated and induces new gene expression profiles as the cells respond to meet the demands of changing microenvironment^3^,^4^. As such, discovering the unique markers expressed in these activated ECs and unraveling their biological functions may help us identify pathways that specifically regulate pathological angiogenesis.

One of the most widely used genetic tools for studying cellular/molecular mechanisms *in vivo* is to induce tissue-specific gene gain- or loss-of-function based on Cre-mediated recombination of loxP sites. Depending on the responding allele, Cre recombinase can either ablate a gene by removing intervening coding sequence flanked by loxP sites (floxed) or activate a gene by excising upstream floxed transcriptional STOP cassettes. Cre-loxP-mediated recombination also enables *in vivo* lineage tracing when used in conjunction with a reporter allele that expresses an indelible marker following excision of STOP cassette. Mouse lines that express Cre under the control of pan-EC enhancer/promoter sequences (for example, Tie1-Cre, Tie2-Cre, Flk1-Cre or VE-Cadherin/Cdh5-Cre) have significantly advanced our understanding of developmental vasculogenesis and angiogenesis^5^,^6^,^7^,^8^,^9^,^10^,^11^. However, these tools target both sprouting and mature vessels, as well as other endothelial-derived cell types, such as the endocardium of the heart or lymphatics (as well as the blood and other tissues)^5^,^6^,^7^,^8^,^9^,^10^,^11^. Interpreting results in a loss- or gain-of -function setting in the context of widespread recombinase activity often makes it difficult to distinguish primary angiogenic effects from secondary effects due to altered cardiovascular development or function. The generation of a novel Cre line that is expressed specifically in sprouting ECs, but not in the endocardium or large vessels, would facilitate more fine-tuned studies of the molecular mechanisms orchestrating sprouting angiogenesis, and would thus allow for more definitive interpretations of *in vivo* mechanistic studies.

One of the most potent inducers of sprouting angiogenesis is hypoxia, which stimulates vascular invasion and growth into oxygen- and nutrient-deficient tissues. The master regulators of hypoxia-induced gene expression are the hypoxia-inducible factor (HIF) family of transcription factors. Under hypoxic conditions, HIF1a induces the expression of several pro-angiogenic molecules. One of these molecules is Apelin (Apln)^12^, a highly conserved 77 amino acid peptide. In addition to Elabela/Toddler^13^,^14^, Apln is an endogenous ligand of the Apln receptor (AplnR or APJ), a seven-transmembrane G protein-coupled receptor^15^. The Apln–AplnR pathway regulates cardiac and vascular function, as well as myocardial cell specification and heart development^16^,^17^,^18^,^19^. In a zebrafish dorsal fin regeneration model, hypoxia-induced Apln expression is required to stimulate ECs proliferation and angiogenesis, a prerequisite for proper organ regeneration^20^. Apln is also a potent chemoattractant for circulating EC progenitors that participate in regenerative angiogenesis after myocardial infarction (MI) in mice^21^. Apln-AplnR signalling functions downstream of the vascular endothelial growth factor A (VEGFA)–VEGF receptor 2 (VEGFR2) pathway, and AplnR is detected in ECs in murine and human tumours^22^,^23^. As Apln expression is activated by hypoxia and VEGF signalling, two potent inducers of sprouting angiogenesis, we hypothesize that Apln may be a molecular marker for sprouting vessels. If true, this characteristic could be exploited for generating tools that specifically target, or label, the angiogenic endothelium.

In this study, we report using the Apln-CreER allele as a novel genetic tool to specifically target sprouting vessels in the developing embryo and adult; such a tool has not been available until now. The Apln-CreER line allows for recombination specifically in sprouting ECs and is useful in gain- and loss-of-function studies *in vivo*. Our work demonstrates that under many pathophysiological conditions, Apln-CreER distinguishes sprouting endothelium from stabilized vessels. This new and unexpected feature of Apln-CreER will be useful in studying the molecular mechanisms of sprouting angiogenesis in developmental biology, tumour angiogenesis, ischaemic diseases and tissue regeneration.

## Results

### Apln-CreER specifically labels angiogenic vessels after ischaemic injury

Recently, we used the Apln-CreER allele to show that subepicardial coronary ECs labelled at embryonic day (E)10.5–E11.5 contribute to the majority of the intramyocardial vascular ECs in the compact myocardium of the developing heart^24^,^25^. During those experiments, we also found that Apln-CreER labelled vascular ECs in other developing organs where active angiogenesis is occurring (Supplementary Fig. 1a–m), an observation consistent with previous work documenting endothelial Apln expression^12^. We performed 5-bromo-4-chloro-3-indolyl β-D-galactopyranoside (X-gal) staining on Apln-LacZ embryos and immunostaining of oestrogen receptor (ESR, as surrogate for APLN) on Apln-CreER tissue sections. We found that vascular ECs robustly express APLN (Supplementary Fig. 2), and these APLN^+^ ECs are actively undergoing proliferation (Supplementary Fig. 3). In addition, Apln-CreER labels vascular ECs specifically, but not endocardial or lymphatic ECs, nor smooth muscle cells (Supplementary Fig. 4). In contrast to the embryos (Supplementary Fig. 1a–m), adult tissues had dramatically fewer Apln-CreER-labelled ECs (Supplementary Fig. 1n–q). This difference may attribute to the fact that Apln expression is enriched in actively sprouting vessels, which are rare in the quiescent, stable vasculature of adult tissues.

**Figure 1:**
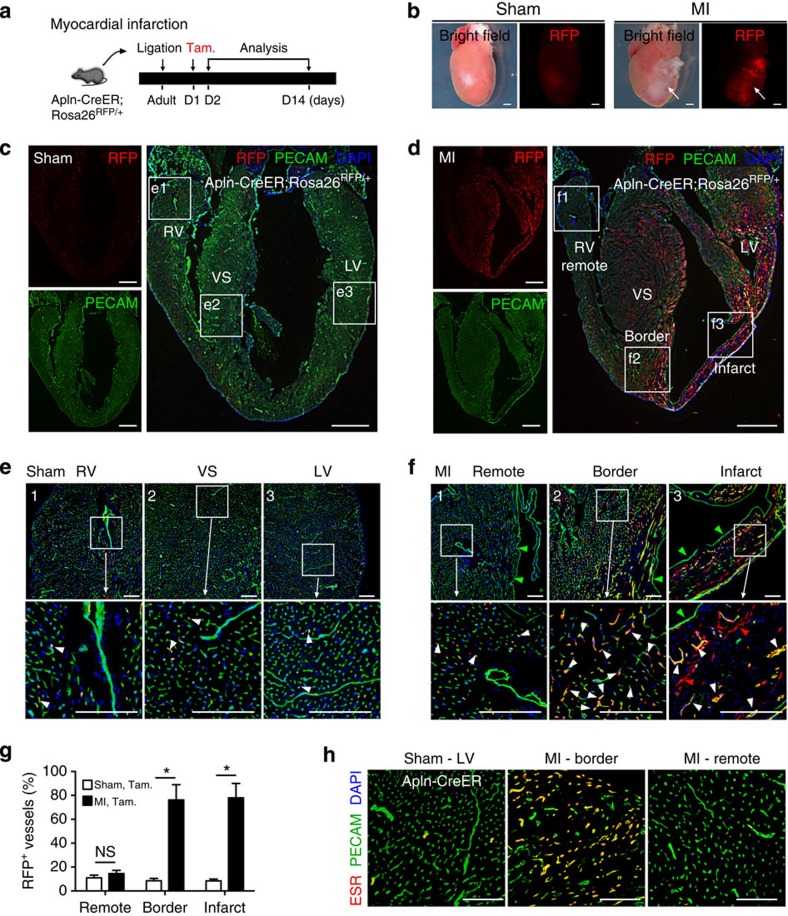
Apln-CreER labels reactivated endothelium in infarcted myocardium. (**a**) Schematic figure showing the strategy for ligation of the LAD coronary artery and genetic labelling of Apln^+^ cells by tamoxifen treatment (Tam.) in Apln-CreER;Rosa26^RFP/+^ adult mice. (**b**) Whole mount view of Apln-CreER;Rosa26^RFP/+^ hearts 14 days after MI or sham. (**c**,**d**) Sections of MI or sham hearts stained with RFP, PECAM and 4',6-diamidino-2-phenylindole (DAPI). Tamoxifen was administered 1 day after MI or sham. (**e**,**f**) RFP^+^ vascular ECs (white arrowheads) in infarct, border or remote zone of MI or sham heart. Green arrowheads denote endocardium; red arrowheads indicate non-vascular RFP^+^ cells. (**g**) Quantification of RFP^+^ vascular ECs. Student’s *t*-test was used to analyse differences and values are shown as means±s.e.m.; *P<0.05, NS, not significant; *n*=5 for MI, 3 for sham. (**h**) Staining of ESR for detection of Apln expression in MI or sham hearts 2 days after MI. LV, left ventricle; RV, right ventricle; VS, ventricular septum. Scale bars, 1 mm (**b**–**d**); 100 μm (**e**,**f**,**h**).

To determine whether Apln is expressed in the adult angiogenic vasculature, we crossed Apln-CreER mice with the Cre-dependent fluorescent reporter line Rosa26^RFP/+^ and subjected them to models of tissue injury that stimulate angiogenesis. Induction of MI (and its sequelae cardiac ischaemia) via ligation of the left anterior descending (LAD) coronary artery stimulates a robust angiogenic response in the surviving coronary vessels to provide new vasculature during recovery. To test whether these angiogenic, activated ECs upregulate the expression of Apln during recovery, we administered tamoxifen following MI in Apln-CreER; Rosa26^RFP/+^ adult mice to label Apln-positive cells (Fig. 1a). Hearts collected 14 days after MI exhibited significantly more red fluorescent protein (RFP) labelling on the left ventricular surface than did the sham-operated controls (Fig. 1b). Immunostaining with RFP and the EC marker platelet endothelial cell adhesion molecule (PECAM or CD31) showed that vascular ECs in the infarcted myocardium and the border zone were efficiently labelled (77.9±12.0% in infarct zone; 76.2±12.7% in border zone), while vessels in remote areas (right ventricle) were infrequently labelled (efficiency 14.5±2.7%; Fig. 1d,f,g). Pulse-chase analysis revealed that RFP^+^ vascular ECs were actively proliferating within the infarcted myocardium (Supplementary Fig. 5). In sham-operated hearts, all examined regions were infrequently labelled by Apln-CreER after tamoxifen administration (Fig. 1c,e,g). By using CreER (detected by an antibody that recognizes the ESR portion of the Cre-ER mutant fusion protein) as a surrogate for APLN^24^, we found that APLN was highly expressed in coronary ECs at day 2 post MI in the border zone, but rare in remote regions or in sham-operated hearts (Fig. 1h). We also observed Apln-CreER activity in the heart after MI without tamoxifen treatment (‘leakiness’; Supplementary Fig. 6a,b), but the amount of Rosa26^RFP^ labelling was negligible compared with post-MI hearts treated with tamoxifen. Immunostaining of LYVE1 (refs 26, 27) confirmed that Apln-CreER-labelled cells are vascular ECs but not lymphatic vascular ECs (Supplementary Fig. 7a). These data demonstrate that Apln expression is initiated in coronary vascular ECs near injured myocardial tissue.

**Figure 2:**
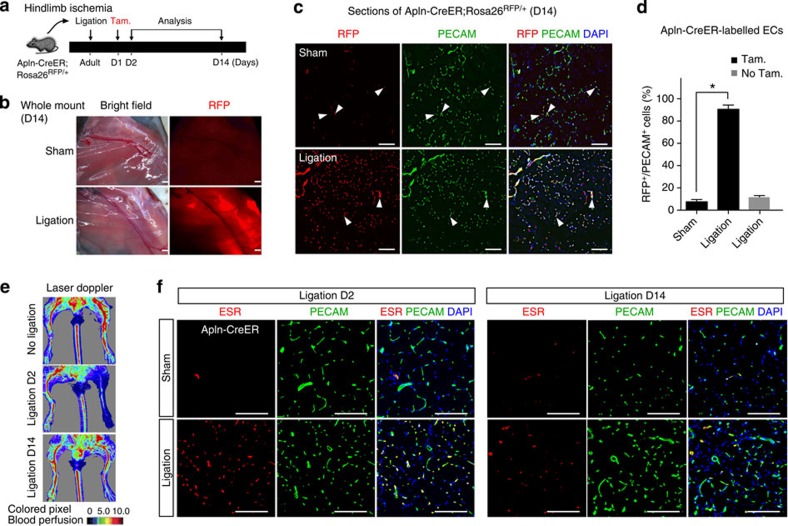
Apln-CreER labels active angiogenesis in the ischaemic hindlimb. (**a**) Schematic figure showing the experimental strategy for ligation of the left femoral artery and labelling of Apln^+^ cells in Apln-CreER;Rosa26^RFP/+^ adult mice. (**b**) Whole mount picture of hindlimb 2 weeks after ligation or sham. (**c**) RFP and PECAM immunostaining of sections from ischaemic and sham-operated hindlimbs. White arrowheads indicate RFP^+^PECAM^+^ cells. (**d**) Percentage of RFP^+^ cells among PECAM^+^ vascular ECs. Analysis of variance was used to analyse differences between groups and values are shown as means±s.e.m.; *P<0.05, *n*=4. (**e**) Laser Doppler perfusion assay of ischaemic limbs. (**f**) Immunostaining of ESR shows Apln-CreER expression in hindlimbs. Scale bars, 1 mm (**b**); 100 μm (**c**,**f**).

To determine whether the reactivation of Alpn-CreER activity was confined to ECs within the neovascularized myocardium, we examined Apln-CreER activity in a hindlimb ischaemia-reperfusion model. We performed femoral artery ligations or sham operations in Apln-CreER;Rosa26^RFP/+^ mice and then administered tamoxifen 1 day after surgery (Fig. 2a). At day 14, we detected CreER-mediated recombination in the tissue adjacent to the femoral artery distal to the ligation (Fig. 2b). Immunostaining showed that a substantial amount of ECs were genetically labelled by Apln-CreER after injury (labelling efficiency in ligated=90.6±3.7% versus sham=7.6±1.9%; *P<0.05) (Fig. 2c,d). These labelled RFP^+^ cells did not differentiate into lymphatic vascular ECs (Supplementary Fig. 7b). A subset of Apln-CreER-labelled RFP^+^ cells were proliferating (Supplementary Fig. 8). On examining the injured limbs after ligation, we observed that local vascular ECs contained a substantial increase in CreER expression, suggesting that the increased RFP labelling of ECs resulted directly from higher Apln expression, rather than the expansion of an initially small population of APLN^+^ ECs (Fig. 2f). Similar to post-MI hearts, in the absence of tamoxifen, we detected a basal level of RFP labelling in ECs of injured hindlimbs, but the degree of labelling was significantly lower than that in the tamoxifen-treated group (Fig. 2d and Supplementary Fig. 6c,d). Interestingly, when blood flow was partially re-established at 2 weeks after injury (Fig. 2e), the number of APLN^+^ vascular ECs decreased (Fig. 2f), consistent with previous work showing that Apln expression is a reliable readout of hypoxia^12^. We next administered tamoxifen 2 weeks after injury, to assay Apln-CreER activity following blood flow recovery (and hence, under less hypoxic conditions). Using this treatment regimen, we observed a significant reduction in Apln-CreER labelling in both MI and hindlimb ischaemia models (Supplementary Figs 9 and 10), although the labelling is still higher than that in sham controls (sham in Figs 1 and 2). Collectively, these results demonstrate that ischaemic-reperfusion injury specifically induces the expression and activity of Apln-CreER in recovering angiogenic vessels within muscle tissue.

### Apln-CreER labels tumour ECs

**Figure 3:**
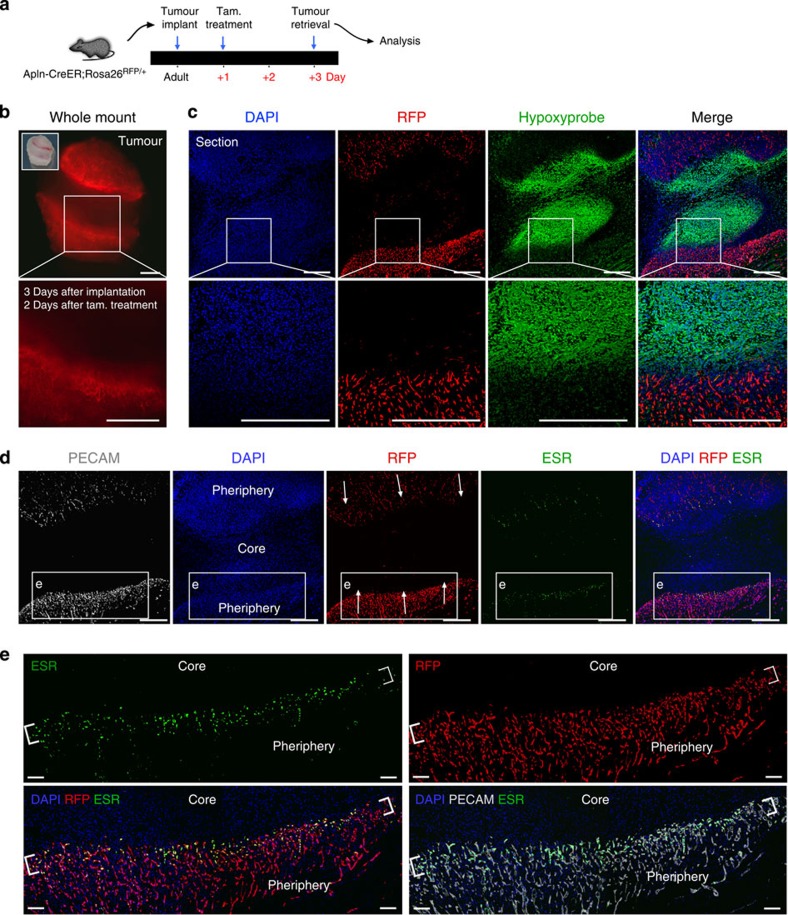
Apln is expressed in sprouting vessels in tumour angiogenesis. (**a**) Strategy of tumour implantation and tamoxifen treatment in Apln-CreER;Rosa26^RFP/+^ mice. (**b**) Whole mount view of implanted tumour at day 3 after implantation. Labelled vessels (RFP^+^) sprout and migrate from surrounding host tissues into the tumour. Tamoxifen was administered 1 day after implantation. (**c**,**d**) Hypoxia was detected by staining of hypoxyprobe on tumour sections. At day 3 after implantation, the tumour periphery was vascularized (RFP^+^PECAM^+^), while the core remains largely non-vascularized (RFP-PECAM-). The tumour core region devoid of blood vessels was highly hypoxic compared with the peripheral region supplied by Apln-CreER-labelled vessels. Apln expression (ESR, green) is enriched in the sprouting angiogenic front. Arrows indicate the direction of migration. (**e**) Magnified images showed elevated APLN (ESR) expression in the sprouting front. The majority of Apln-CreER lineage-traced vessels (RFP^+^) do not continue to express APLN, while tip cells actively express APLN (brackets, RFP^+^ESR^+^). Each figure is merged from two split pictures. Images are representative of six individual samples. Scale bars, 500 μm (**b**,**c**,**d**); 100 μm (**e**).

The microenvironment of a solid tumour is highly hypoxic, and dynamic ECs migrate outward from existing vessels in response to environmental cues until they form new perfused and stable vessels^28^. Hence, given our finding that Apln-CreER labels sprouting endothelium but not mature quiescent vessels, we aimed to test whether this genetic tool could be used to study tumour angiogenesis. Well-established tumour cell lines (Lewis lung carcinoma (LLC), TC-1, E.G7 and Hepa1-6) were embedded in Matrigel and implanted subcutaneously into the groin of adult Apln-CreER;Rosa26^RFP/+^ mice (Supplementary Fig. 11). Tamoxifen was administered after tumour implantation to label murine Apln-expressing ECs and their descendants. Mice were treated with tamoxifen on post-implantation day 1 and tumour samples were collected 2 days later (3 total days after implantation) (Fig. 3a). At this early time point, only the peripheral edges of the tumour should contain sprouting ECs and the tumour core should be devoid of host-derived ECs. Apln-CreER-labelled cells (RFP^+^) were restricted to the tumour periphery, with few RFP^+^ cells observed in the core (Fig. 3b). Immunostaining confirmed that the RFP^−^ tumour core was severely hypoxic, while the RFP^+^ tumour periphery was more normoxic (Fig. 3c). Immunostaining for PECAM demonstrated that these Apln-CreER-derived RFP^+^ cells are in fact ECs, and that Alpn-CreER labels the vast majority of tumour-induced ECs (Fig. 3d). Apln-CreER does not label the lymphatic vascular ECs in this setting (Supplementary Fig. 7c). In addition, we detected that a subset of these RFP^+^ ECs are proliferating in tumour samples (Supplementary Fig. 12). We again used ESR antibody to detect CreER as surrogate for active APLN expression^24^. Intriguingly, a more detailed examination of the tumour hypoxic–normoxic interface revealed that ECs in the sprouting angiogenic front orienting towards the hypoxic tumour core were enriched for ALPN expression (ESR^+^), while ECs in the more normoxic areas showed reduced ALPN expression (Fig. 3d,e). A reasonable explanation suggests that the ECs closest to the hypoxic core are exposed to elevated angiogenic signals, such as VEGF. Accordingly, these sprouting cells, which display a more dynamic, tip-cell-like phenotype, are enriched for APLN. Conversely, those ECs in the more normoxic areas behave similar to stalk cells and present with decreased APLN levels. Here, by pulse-chase analysis, we showed that most tumour vessels present at day 3 are descendants of Apln-expressing cells at day 1 after tumour implantation (when tamoxifen was administered). At day 3, the RFP^+^ cells that are distal to the angiogenic growth front are descendants of Apln-expressing cells labelled at day 1, although they no longer actively express APLN (as detected by ESR) (Fig. 3e). However, those RFP^+^ cells in the angiogenic growth front also robustly express APLN (ESR^+^). The significant contribution of the early Apln-CreER lineage to the tumour vasculature several days later, combined with APLN’s restricted expression in the angiogenic front at that later stage, suggested that APLN is enriched in, and therefore Apln-CreER labels, sprouting angiogenic ECs. Furthermore, collection of tumour samples after even as little as 24 h following tamoxifen induction showed a significant overlap between the active APLN expression (ESR^+^) and the Apln-lineage (RFP^+^; Supplementary Fig. 13a,b). In addition to sprouting and migration, another characteristic behaviour of angiogenic ECs is proliferation. Indeed, Alpn-lineage ECs not only sprout and migrate but they also proliferate, which explains the broad contribution of the day 1 lineage to the day 3 tumour vasculature (Supplementary Fig. 13c). Owing to the profound angiogenic activity of the allele at this early stage of tumorigenesis (Fig. 3 and Supplementary Fig. 13), we next analysed Alpn-CreER activity in the setting of well-established mature tumours.

**Figure 4:**
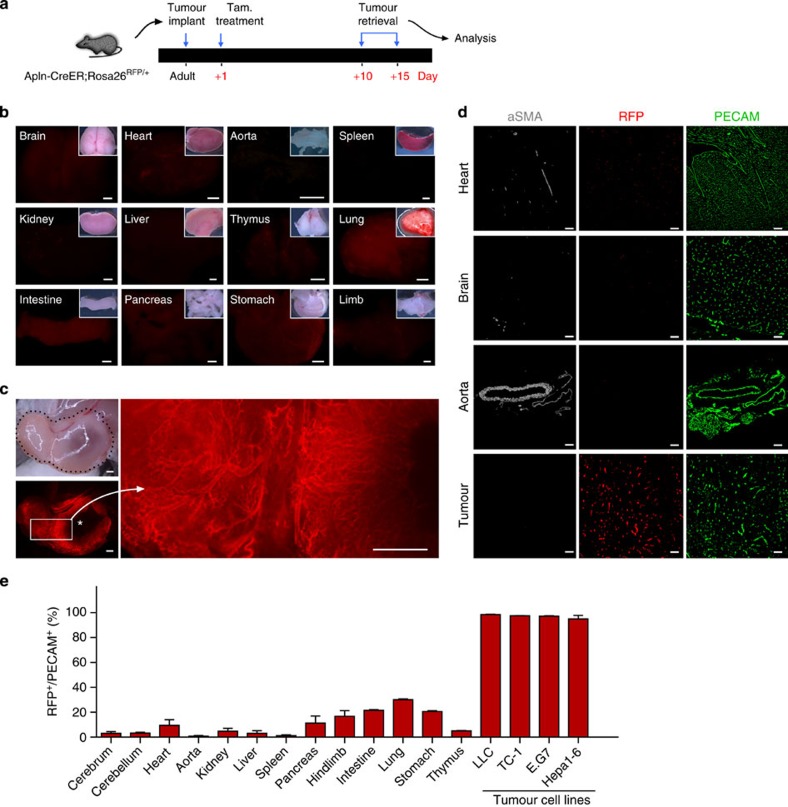
Lineage tracing of Apln^+^ cells in organs and tumours. (**a**) Strategy of tumour implantation and tamoxifen treatment on Apln-CreER;Rosa26^RFP/+^ mice. Tumours were retrieved at 10 to 15 days after implantation with obvious growth in size and processed for quantification of Apln-labelled vessels. (**b**) Whole mount pictures of organs or tissues from Apln-CreER;Rosa26^RFP/+^ host mouse. (**c**) Whole mount pictures of implanted tumour (black dotted line) within host tissue. Magnified figures showed Apln-labelled tumour vessels. Asterisk indicates necrotic region inside tumour devoid of blood vessel. (**d**) Staining of genetic marker RFP and vascular EC marker PECAM, and smooth muscle marker α-smooth muscle actin (aSMA) on sections of the heart, brain, aorta and tumour tissue. (**e**) Quantification of RFP^+^ cells in PECAM^+^ vascular ECs of different organs and tumours. Values are shown as means±s.e.m.; *n*=3–5. Scale bars, 1 mm (**b**,**c**); 100 μm (**d**).

Tumours were implanted as above, with tamoxifen treatment at days 1, and tumours were collected for analysis at day 10 to 15, when it’s mass was greatly expanded (Fig. 4a). In addition to the tumours, we collected various tissues and organs to assess the extent of vessel labelling (*n*=3–4 for each organ examined). Whole-mount examination and immunofluorescent staining with quantification revealed a large number of host-derived RFP^+^ ECs in the tumour tissue, yet significantly fewer RFP^+^ ECs were detected in other organs or tissues (Fig. 4b–e and Supplementary Fig. 14). RFP^+^ tumour vessel ECs were infrequently surrounded by vascular smooth muscle cells, a hallmark of the unstable, immature vessels associated with cancer (Fig. 4d). Similar results were obtained using the Rosa26^mTmG/+^ reporter allele^29^ (Supplementary Fig. 15). To determine whether these Apln-CreER-labelled vessels were functional, we injected fluorescein-conjugated BS1-lectin into murine circulation via the inferior caval vein. After collecting tumour samples, we found that the RFP^+^ ECs in tumours are also BS1-lectin positive, suggesting that the RFP^+^ vessels are patent, lumenized vessels capable of conducting blood flow (Supplementary Fig. 16). Overall, these experiments demonstrate that Apln-CreER can be used to specifically label the tumour neovasculature.

**Figure 5:**
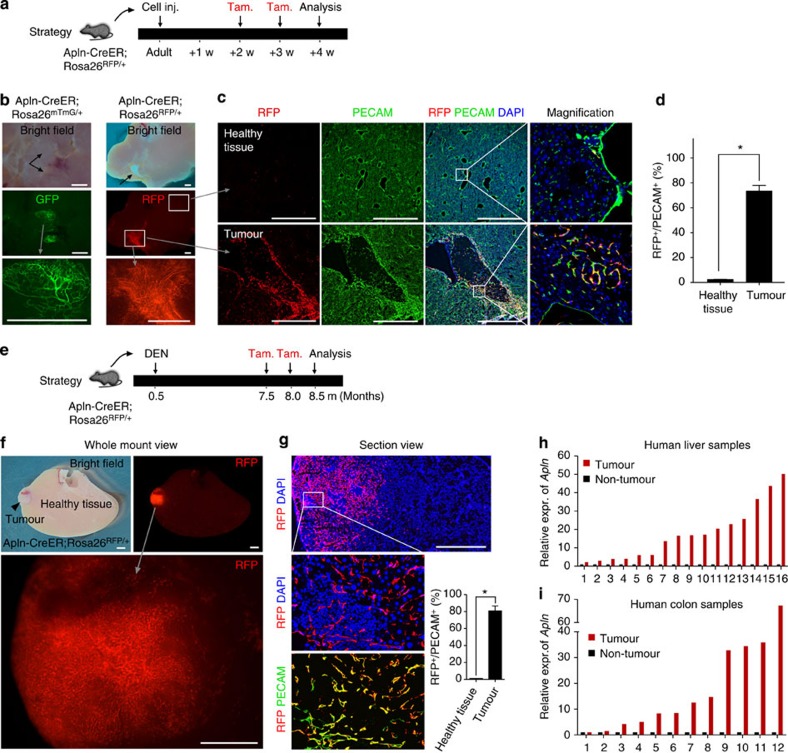
Apln-CreER labels active angiogenesis in orthotopic and spontaneous tumour models. (**a**) Strategy of orthotopic tumour model establishment and tamoxifen treatment strategy. (**b**) Whole mount view of liver orthotopic tumours based on Apln-CreER;Rosa26-Reporter mice. Black arrows indicate tumours. The grey arrows and boxed areas in the middle panels are magnified below. (**c**) Immunostaining of RFP, PECAM and DAPI (4',6-diamidino-2-phenylindole) on tumour and healthy tissue sections. (**d**) Quantification of RFP^+^ vessels. Student’s *t*-test was used to analyse differences and values are shown as means±s.e.m.; *P<0.05; *n*=4. (**e**) Strategy of *in situ* tumorigenesis by DEN treatment. DEN was injected at P0.5m (postnatal half month) and tamoxifen was administered at P7.5m and P8m, and tumours were collected at P8.5m. (**f**) Whole mount view of DEN-induced liver tumour model. The tumour (black arrowheads) is enriched for Apln^+^ vessels (RFP^+^). Representative of at least five individual mice. (**g**) Immunostaining of RFP, PECAM and DAPI on sections of DEN-induced tumour sample. Student’s *t*-test was used to analyse differencesand values are shown as means±s.e.m.; *P<0.05; *n*=5. (**h**,**i**) Tumour and healthy tissue from the liver or colon of the same patient were collected (*n*=16 for liver and *n*=12 for colon). *Apln* mRNA expression was measured by quantitative reverse transcription PCR and normalized to *β-Actin* mRNA. Relative *Apln* expression in non-tumour tissue surrounding tumour sample was assigned a value of 1. Expr., expression. Scale bars, 1 mm.

To assay Apln-CreER labelling efficiency in more physiologically relevant tumour angiogenesis models, we generated both orthotopic and chemically induced cancers in the liver. Orthotopic tumours were established by injecting hepatic cancer cells (Hepa 1–6) directly into the liver of Apln-CreER;Rosa26^RFP/+^ mice. APLN^+^ vessels were detected in ECs of the orthotopic tumour (Supplementary Fig. 17). We also employed a model of *in situ* spontaneous tumorigenesis by applying the genotoxic carcinogen diethylnitrosamine (DEN) to Apln-CreER;Rosa26^RFP/+^ mice, which produces hepatic cancers ~8 months post injection^30^. In both cases, tamoxifen was administered two times before tissue retrieval (Fig. 5a,e). We found that Apln-CreER labels ECs in both the orthotopic and chemically induced liver tumour models, while ECs in adjacent healthy tissue were RFP^−^ (Fig. 5b,c,f,g). Immunostaining and quantification confirmed that the number of labelled vessels was significantly elevated in these tumours (Fig. 5c,d,g). Furthermore, mesenteric tumours were also found in some DEN-injected animals, and their associated vasculature was also strongly labelled by Apln-CreER (Supplementary Fig. 18). Thus, Alpn-CreER labelled the vasculature in both physiological and xenograft tumour models, while inducing less recombination in the vascular beds of other healthy tissues.

Next, we obtained matched tumour and para-tumour tissue samples from patients with hepatocellular carcinoma and colon cancer (Supplementary Table 1). We found that in all cases, APLN expression was significantly elevated in both liver and colon tumours compared with surrounding non-tumour tissue from the same patient (Fig. 5h,i). These results mirror the increased Apln expression we observed in angiogenic ECs in our murine tumour models, and together they suggest that increased Apln expression during angiogenesis may be a clinical hallmark of human carcinogenesis.

### Apln-CreER-mediated cell ablation in tumour vessels

**Figure 6:**
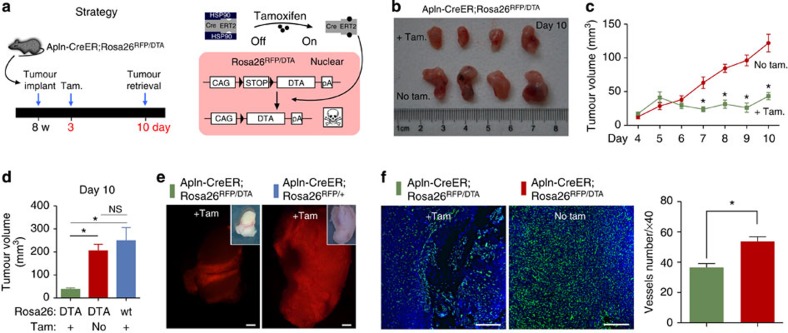
Apln-CreER-mediated genetic ablation of tumour vessels. (**a**) Schematic figure showing the strategy underlying the Apln-CreER tumour EC ablation model. Tam., tamoxifen; DTA, diphtheria toxic fragment A. (**b**) Picture of tumours from Apln-CreER;Rosa26RFP/DTA mice treated with tamoxifen (+Tam.) or PBS (No Tam.). (**c**) Quantification of tumour volume (length × width^2^/2 (mm^3^)) at various times after implantation. Student’s *t*-test was used to analyse differences and values are shown as means±s.e.m.; *P<0.05; *n*=12 for each time point. (**d**) Quantification of tumour volume of three different groups as indicated. wt, wild-type allele (no DTA) of the Rosa26 locus. Student’s *t*-test was used to analyse differences between groups, and values are shown as means±s.e.m.; *P<0.05; NS, not significant. *n*=4–6. (**e**) Whole mount view of RFP^+^ tumours. (**f**) Vessel detection by PECAM staining and quantification of vessel number in different groups. Student’s *t*-test was used to analyse differences, and values are shown as means±s.e.m.; *P<0.05; *n*=3–4. Scale bars, 1 mm (**e**), 0.5 mm (**f**).

As tissue-specific expression of Cre recombinase facilitates analyses of gene function, as well as cell lineage tracing, we hypothesized that Apln-CreER could be employed for analysis of the cellular or molecular mechanisms regulating tumour angiogenesis. As a proof of principle, we used diphtheria toxin (DT) to conditionally ablate Apln-CreER-expressing angiogenic ECs in murine tumour models. DT is composed of two subunits, A and B. On binding to its receptor and internalization, subunit A (DTA) inactivates the elongation factor 2, terminating protein synthesis and ultimately causing cell death^31^. By crossing the Apln-CreER allele into a compound heterozygous Cre-inducible DTA (Rosa26-DTA)^32^, Rosa26-RFP reporter background, we generated Apln-CreER;Rosa26^DTA/RFP^ mice. Owing to the presence of a loxP-flanked transcriptional stop cassette upstream of the DTA complementary DNA, DTA is not expressed in the absence of tamoxifen (similar to the loxP-stop-loxP RFP reporter allele). Following tamoxifen treatment, there is simultaneous recombination of the DTA and RFP alleles (Fig. 6a). Tamoxifen was injected 2 days after tumour implantation, allowing for a robust angiogenic response from the host tissue to provide nutrition and oxygen to the growing tumour. Tumours were collected 1 week later to analyse the effects of Apln-CreER-mediated EC ablation on tumour growth and vascularization (Fig. 6a). In the tamoxifen-treated group, tumours were significantly smaller compared with the PBS-treated controls (Fig. 6b). By monitoring the dynamics of tumour growth in Apln-CreER;Rosa26^DTA/RFP^ mice, we found that tamoxifen-treated tumours failed to expand, while the tumour volume in the control group steadily increased (Fig. 6c). To exclude the potential confounding effects of tamoxifen on the growth of tumour cells and/or vascular ECs, we performed the experiment in Apln-CreER;Rosa26^RFP/+^ mice as a control. Tumour volume was only significantly reduced in the presence of the DTA allele, demonstrating that the observed tumour growth retardation was independent of tamoxifen treatment (Fig. 6d). There was reduced RFP labelling and vessel density in the tumours of Apln-CreER;Rosa26^DTA/RFP^ mice (Fig. 6e), which was associated with regions of tissue necrosis (Fig. 6e,f). Other tissues and organs were analysed for potential off-target effects by Apln-CreER-mediated DTA expression. After histological examination (haematoxylin and eosin staining) and quantification of vascular density, we did not observe defects in the non-cancerous tissue of several other organs (Supplementary Figs 19 and 20), suggesting that a small degree of vascular EC death in other organs can be well tolerated. The above findings demonstrate that Apln-CreER specifically targets tumour vascular ECs without significantly affecting other organs or tissues.

### Apln-CreER-mediated *VEGFR2* gene ablation

**Figure 7:**
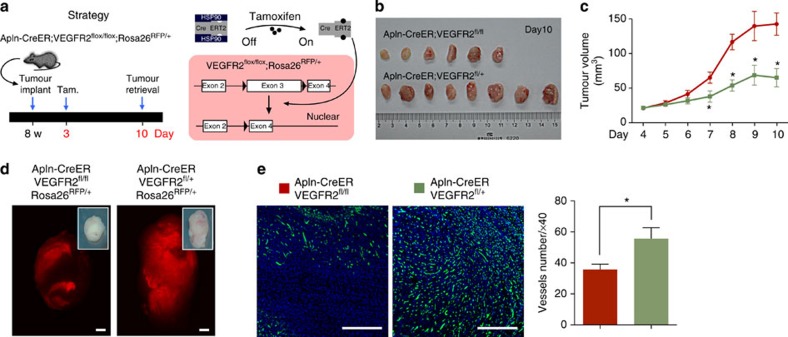
Apln-CreER-mediated genetic ablation of VEGFR2. (**a**) Schematic showing the strategy and principle of induced VEGFR2 ablation in Apln^+^ cells. Tam., tamoxifen treatment. (**b**) Picture of tumours from Apln-CreER;VEGFR2^fl/fl^ and littermate controls Apln-CreER;VEGFR2^fl/+^ mice. (**c**) Quantification of tumour volume (length × width^2^/2 (mm^3^)) at different times following implantation. Student’s *t*-test was used to analyse differences and values are shown as means±s.e.m.; *P<0.05; *n*=6–8 for each time point. (**d**) Whole mount view of RFP^+^ tumours. (**e**) Vessel detection by PECAM staining and quantification of vessel number in different groups. Student’s *t*-test was used to analyse differences and values are shown as means±s.e.m.; *P<0.05; *n*=6. Scale bars, 1 mm (**d**); 0.5 mm (**e**).

The VEGF-VEGFR2 signalling pathway plays a key role in vascular development and disease, and the VEGF axis has emerged as a central therapeutic target to combat tumour angiogenesis^30^,^33^,^34^. However, VEGF signalling has never been specifically inhibited in sprouting ECs *in vivo*. To accomplish this, we crossed Apln-CreER;Rosa26^RFP/+^ mice with mice harbouring a floxed allele of the *VEGFR2* gene (*VEGFR2*^*fl*^)^35^,^36^, generating Apln-CreER;VEGFR2^fl/fl^;Rosa26^RFP/+^ mice, in which we simultaneously deleted VEGFR2 and performed lineage tracing of mutant ECs and their descendants (Fig. 7a). Two days after tumour implantation, we induced Cre-mediated recombination by tamoxifen treatment and examined tumour growth 7 days later. Ablating VEGR2 via Apln-CreER significantly reduced tumour volumes as compared with littermate controls (Fig. 7b,c). In addition, the presence of RFP^+^ vessels was dramatically decreased in tumours from Apln-CreER;VEGFR2^fl/fl^;Rosa26^RFP/+^ mice (Fig. 7d). In addition, immunostaining for PECAM revealed that the total number of vascular ECs in the tumours of Apln-CreER;VEGFR2^fl/fl^;Rosa26^RFP/+^ mice was lower than that in control mice (Fig. 7e). In contrast to the tumour tissue, other organs from Apln-CreER;VEGFR2^fl/fl^ mice showed no detectable difference in vascular density (Supplementary Fig. 21). As such, Apln-CreER can be considered as a valuable, novel tool for performing genetic loss-of-function studies of tumour angiogenesis.

## Discussion

In this study, we characterized the Apln-CreER allele and found that its activity distinguishes actively angiogenic vessels from the stable and quiescent endothelium. By performing genetic lineage tracing studies during embryogenesis as well as in adulthood under the pathophysiological conditions of ischaemia and tumour angiogenesis, we demonstrate that Apln-CreER mediates robust recombination of loxP-flanked gain- and loss-of-function alleles specifically in sprouting angiogenic vascular ECs. Thus, this allele will enable the dissection of the cellular and molecular mechanisms that regulate angiogenesis during embryonic development, tumour angiogenesis, tissue ischaemia and regeneration.

Our studies first examined coronary vessel formation in the heart after MI to investigate regenerative angiogenesis. Previously, we showed that Apln-CreER labels subepicardial vascular ECs in the heart and that the descendants of these cells give rise to most intramyocardial vascular ECs^24^. We hypothesized that this response was triggered by an elevated demand for oxygen and nutrients after the dramatic increase in muscle mass that occurs during fetal heart development^37^. Conversely, in normal adult heart, there is limited, if any, tissue growth and the coronary vessels supply adequate oxygen and nutrients to the muscle. In this context, Apln expression and thus Apln-CreER activity in the coronary ECs is low.

However, pathological insults such as MI initiate a cascade of events that fundamentally alter the heart and the resident vasculature. One of the detrimental effects of an MI is the generation of a large area of hypoxic tissue within the heart. This, in turn, initiates the expression of various hypoxia inducible/HIF1α-responsive genes within ECs, including *Apln*. The activation of this angiogenic gene programme and the subsequent switch of quiescent ECs to an actively angiogenic phenotype precedes the neovascularization of the compromised myocardium. This ischaemia/injury-induced upregulation recapitulates the pattern of Apln expression seen in the developing sprouting vasculature during embryogenesis. By imploying Apln-CreER, we were able to label most of the ECs (and their descendants) that upregulated Apln expression following MI. As the coronary vasculature re-entered a more quiescent state at ~2 weeks post MI (compared with that at day 2 post MI), Apln expression and CreER activity was significantly reduced. Thus, the Apln-CreER allele allowed us to map the fate of activated, angiogenic coronary ECs specifically during the hypoxic phase of MI recovery, but its activity was reduced on the re-establishment of a functional vasculature. This lineage-tracing property could be used to investigate the cellular origin of newly formed vessels and identify the molecular mechanisms that regulate sprouting angiogenesis during injury and repair processes. However, the utility of this allele is not limited to injury-induced models of angiogenesis.

Blood vessels are also indispensible for tumour growth and metastasis. In many cancers, the degree of vascularization is inversely correlated with patient survival^34^. In addition, many solid tumours require vascular invasion as a prerequisite for metastasis. In most cases of solid tumour expansion, ECs from vessels in the surrounding area remodel their microenvironment, degrade matrix proteins, lose connections with smooth muscle cells and pericytes, and dynamically sprout and migrate into nearby tumour tissue, supporting its continued growth^38^. This is a well-studied mode of tumour vessel formation and many anti-angiogenic drugs are approved for (or being developed to target) tumour angiogenesis^39^. However, to refine therapeutic approaches targeting EC growth, we require a deeper understanding of the molecular regulatory networks that govern tumour angiogenesis. Our Apln-CreER allele provides a unique tool for selectively targeting tumour angiogenesis and it may aid investigations into both the cellular and molecular mechanisms of tumorigenesis in murine models. Our work shows that although Apln-CreER is not active in the majority of blood vessels in a healthy adult animal, in multiple tumour models it clearly labels a substantial percentage of the tumour vasculature. In some cases, it labelled the tumour vascular endothelium with near total efficiency, strongly suggesting that Apln itself may be an attractive target for restricting pathologic angiogenesis, tumour growth and potentially metastasis.

Although anti-angiogenic therapies are now an important therapeutic strategy in oncology, limitations to this approach have emerged during preclinical efficacy studies. Notably, some cancers are resistant to anti-VEGF therapy and, even at its most effective, this class of drugs only prolong patient survival for months (rather than years). This is attributed to a tumour’s ability to evolve alternative modes for stimulating vascular growth, which ultimately allow the disease to rebound or even progress^34^,^38^. Some hypothesize, quite cogently that as tumours secrete multiple pro-angiogenic molecules (FGFs, IL8, Dll4, PlGF, Ang and so on), targeting only the VEGF signalling axis may explain the lower-than-expected efficacy in the clinic^40^. Paradoxically, some studies show that anti-angiogenic therapy actually increases metastasis in mice treated with VEGF inhibitors^41^, but the role of VEGF inhibition remains to be determined^42^,^43^. Further complicating the issue, anti-angiogenic treatments also affect physiological angiogenesis, causing substantial side effects (such as hypertension, thrombosis, gastrointetestinal perforation, renal failure and congestive heart failure, among other complications)^38^,^44^,^45^,^46^,^47^. Accordingly, future discoveries that focus on the mechanisms regulating tumour angiogenesis will probably improve or even supplant current treatment regimens.

Thus, markers that distinguish normal endothelium from the tumorigenic neovasculature are a prerequisite for identifying novel, tumour EC-specific therapeutic targets that spare healthy vessels in other organs and tissues from off-target effects. Our study defines Apln as a specific marker of tumorigenic sprouting ECs and demonstrates the utility of Apln-CreER for both lineage tracing and genetic manipulation of the tumour vasculature.

## Methods

### Mice

All mice were used in accordance with the guidelines of the Institutional Animal Care and Use Committee of the Institute for Nutritional Sciences, Shanghai Institutes for Biological Sciences, Chinese Academy of Sciences. Apln-CreER, Apln-LacZ, VEGFR2 flox, Rosa26-RFP, Rosa26-mTmGand Rosa26-DTA mice were described previously^12^,^24^,^29^,^32^,^36^,^48^,^49^ and maintained on a C129/C57BL6/J-mixed background. Tamoxifen (Sigma, T5648) was dissolved in corn oil (20 mg ml^−1^) and introduced by oral gavage at the indicated time (0.1–0.15 mg tamoxifen per gram mouse body weight).

### Myocardial infarction

MI was performed as previously described^50^. Briefly, 8~20-week-old adult male or female mice were anaesthetized with isoflurane in an induction chamber. After mice were nonresponsive to external stimuli, their chests were shaved with a hair clipper and the skin was disinfected with iodine. The mice were ventilated and anaesthetized with trachea cannula, connected to a respiratory machine (Harvard Apparatus) and an anaesthetic gas machine (Harvard Apparatus). Their respiration was maintained at about 120~140 breaths per minute. After mice were secured on a heating pad to maintain their body temperature at about 37 °C, a longitudinal incision of 1–1.5 cm was made in the sternum between the third and fourth ribs. The heart was exposed through a left thoracotomy and a retractor was used to ensure a good field of view. The LAD coronary artery was occluded with a suture at the upper 1/3 location of LAD coronary artery. After successful ligation, the chest cavity was closed by bringing together the third and fourth ribs with one 5–0 suture, and then the thorax and the skin were sutured closed layer by layer and disinfected with idodine. Similarly, the shame-operated control mice were anaesthetized for thoracotomy, but without LAD ligation. The anaesthetic gas machine was turned off and then the trachea cannula was removed until mice displayed autonomous respiration. For better postoperative recovery, mice were kept warm for several hours.

### Unilateral hindlimb ischaemia model

The hindlimb ischaemia model was performed as previously described^51^. Briefly, mice were anaesthetized with 10% chloral hydrate (4 μl g^−1^, intraperitoneally (i.p.)). Once they were nonresponsive to external stimuli, mice were placed on a heated pad to maintain a core temperature of 37 °C. Mice were placed in a supine position and their hindlimbs were extended and fixed in place by adhesive tape. Abdominal fur was removed and the skin was disinfected with iodine and 75% alcohol before surgery. An incision of skin was made parallel to the femur by ophthalmic scissors and forceps. Subcutaneous fat tissue and epimysium was dissected to reveal the underlying femoral artery. The femoral artery was separated from the femoral vein at the groin region and was ligated by a 5–0 suture. The incision on epimusium and the skin was stitched and disinfected with iodine before mice were revived.

### Laser Doppler blood perfusion

A Laser Doppler blood flow imager (Moor Instruments) was used to assess the extent of blood-flow (perfusion) restoration in mice after surgery as previously described^52^. Mice were anaesthetized and the femoral artery was ligated as explained in the hindlimb ischaemia procedure. Mice were placed on a black, non-reflective light-absorbing cloth (supplied by the Laser Doppler system) in the supine position. The hindlimb was placed in the measuring field and image data were acquired. To account for variables, including ambient light and temperature, blood flow was calculated in the foot as a ratio of the ischaemic to the non-ischaemic leg of the same mouse. Mice showing <60% blood flow reduction were excluded from this study.

### Tumour cell lines and implantation model

Mouse lung tumour cell line TC-1, lymphoma cell line E.G7, hepatocarcinoma cell line Hepa1-6 and LLC cell line were bought from the cell bank of Shanghai Institutes of Biological Sciences. TC-1 and E.G7 cells were maintained in RPMI-1640 medium (HyClone, SH30809.01B) with 10% fetal bovine serum (Invitrogen, 10099141), and LLC and Hepa1-6 cells were cultured in DMEM/HIGH GLUCOSE medium (HyClone, SH30243.01B) containing 10% fetal bovine serum. Tumours were obtained by inoculating 2 × 10^6^ cells either subcutaneously or in the liver. For the subcutaneous tumour model, cells were dissolved in 100 μl RPMI-1640 or DMEM medium and gently mixed 1:1 (volume ratio) with Matrigel (BD 356237) immediately before injection (200 μl total volume per injection), and cells were injected in the groin region of mice. For the orthotopic tumour model in the liver, the total injection volume was kept under 40 μl and the volume ratio of cells to Matrigel was 1:2~1:3. To perform liver *in situ* injection, mice were anaesthetized and ventilated just as the MI procedure described above; cells were injected in the left lobe of mouse liver. For lineage tracing, Tamoxifen (Sigma) was administered to induce Cre-mediated LoxP recombination. In the subcutaneous tumour model, tamoxifen was given 1 day after inoculation. In the liver orthotopic tumour model, tamoxifen was administered once at week 2 and once at week 3 after inoculation. The subcutaneous and orthotopic tumour tissues were dissected either 10–15 days or 1 month after inoculation.

### DEN induced hepatocarcinoma

Two-week-old newborn pups were i.p. injected with 25~50 mg kg^−1^ N-nitrosodiethylamine (DEN) (Sigma N0258), then orally gavaged with 5 mg tamoxifen at 7 and 7.5 months after DEN injection. Mice were killed 8 months after the initial DEN injection and the livers were harvested and examined for the presence of hepatocarcinoma formation. EC labelling within the tumour was determined by visualization of the recombined Cre-reporter locus (Rosa26^RFP/+^ or Rosa26^mTmG/+^). After whole-mount images of the samples were recorded, the livers were fixed, dehydrated and embedded in OCT for frozen sectioning and subsequent immunostaining.

### Human tumour sample

Patient hepatocellular carcinoma samples were collected after informed consent according to an established protocol approved by the Ethics Committee of Eastern Hepatobiliary Surgery Hospital. Samples were obtained from ten adult males and six adult females with age ranging from 35 to 73 years. Of the 16 samples, 7 had macroscopically or microscopically visible vessel invasion (6 males and 1 female). Human colon cancer samples were collected at the Zhongshan Hospital after we received patients’ informed consent approving further analysis of their samples. All procedures were reviewed and approved by the Institutional Review Board of Institution for Nutritional Sciences (Protocol number E-2011-03). Human colon cancer samples were obtained from ten adult males and two adult females with age ranging from 37 to 80 years. Of the 12 samples, 8 were obtained from the rectum (1 female and 7 male) and 4 samples were collected from the colon (1 female and 3 male). Half of the patients presented with lymph node metastasis and two patients also possessed distal metastasis. Detailed information was shown in Supplementary Table 1.

### X-gal staining

Specimens were fixed in LacZ fix solution (0.2% glutaraldehyde, 5 mM EGTA, and 100 mM MgCl_2_ in PBS) for 15–60 min, depending on size. After washing with LacZ wash buffer (2 mM MgCl_2_, 0.01% sodium deoxycholate, 0.02% NP-40 in 100 mM sodium phosphate buffer) three times at 30 min per wash, samples were stained in LacZ staining buffer (1 mg ml^−1^ X-gal in LacZ wash buffer) at 37 °C overnight. Tissues were washed with LacZ wash buffer three times and then fixed with 4% paraformaldehyde (PFA) before imaging.

### Immunostaining

Organs and tumours were collected in PBS and fixed in 4% PFA (sigma) at 4 °C for 1–3 h, depending on tissue size. After washing three times in PBS, tissues were equilibrated in 30% sucrose (Sigma) at 4 °C overnight followed by OCT (Sakura) embedding and frozen sectioning (Thermo HM525 cryosection machine). For circulation perfusion assay, 400 μl fluorescein-labelled Griffonia (Bandeiraea) Simplicifolia Lectin I (FITC BS1 lectin, Vector Lab, FL-1101-5, diluted in PBS 1 μg μl^−1^) was injected into the inferior caval vein of pre-anaesthetized tumour-carrying mice 1 h before killing, and then tumour samples were dissected and processed as stated above. Cyrosections of 10 μm thickness were collected on slides and incubated in blocking solution (5% normal donkey serum in PBS with 0.1% Triton X-100) for 30 min at room temperature and then incubated with the primary antibodies diluted in 2.5% normal donkey serum in PBS with 0.1% Triton X-100 overnight at 4 °C. Primary antibodies were commercialized reagents: PECAM (BD Pharmingen, 553370, 1:200), SMA (Sigma, F3777, 1:200), ESR (Abcam, ab27595, 1:1), VE-Cadherin (R&D, AF1002, 1:100), LYVE1 (Abcam, ab14917, 1:100), RFP (Rockland, 600-401-379, 1:1,000), GFP (Invitrogen, A21311, 1:100), BRDU (Abcam, ab6326, 1:100), EDU (Invitrogen, C10337, C10340), Hypoxyprobe (Hypoxyprobe Inc., HP3-100Kit). Signals were developed with Alexa fluorescent antibodies (Invitrogen) and nuclei were visualized by 4',6-diamidino-2-phenylindole (Vector labs) staining. Images were obtained on an Olympus confocal microscope (FV1200).

### Hypoxia detection

Tumour tissue hypoxia was detected by Hypoxyprobe-1 Omni Kit (Hypoxyprobe Inc.), according to the manufacturer’s protocol. Mice were i.p. injected with 60~120 mg kg^−1^ pimonidazole HCl (25 mg ml^−1^ in 0.9% NaCl). At 60 min after injection, tumours were collected and fixed in 4% PFA overnight, followed by washing in PBS, and then equilibrated in 30% sucrose and embedded in OCT for frozen sectioning. Hypoxyprobe was visualized by rabbit anti-pimonidazole antisera and Alexa donkey anti-rabbit antibody.

### Quantitative reverse-transcription PCR

Quantitative reverse-transcription PCR was performed as previously described^50^. Total RNA was extracted from human tumour and para-tumour tissue with Trizol, according to the manufacturer’s instructions (Invitrogen) and converted to cDNA by M-MLV reverse transcriptase (Progema, M170A). SYBR Green qPCR master mix (Applied Biosystems) and the Applied Biosystems 7500 Real-Time PCR System were used to perform quantitative PCR. β-Actin was used as internal control. The primers for human Apln and β-actin are listed below: Apln 5′- TCCCAAATCGGTTCTAGGTC -3′ (forward), 5′- CCTGTAAGTGGGCTGGATTT -3′ (reverse); β-actin 5′- GATCATTGCTCCTCCTGAGC -3′ (forward), 5′- ACTCCTGCTTGCTGATCCAC -3′ (reverse).

### Cell ablation and VEGFR2 loss of function

Eight to 20-week-old male or female Apln-CreER;Rosa26^DTA/RFP^ mice were bred for Apln^+^ cell ablation; Apln-CreER;VEGFR2^flox/flox^;Rosa26^RFP/+^ mice were used for *VEGFR2* gene ablation. Two days after subcutaneous injection of TC-1 cells, mice were treated with 5 mg tamoxifen. Mice were killed 10 days after TC-1 inoculation, and tumour tissue as well as other organs or tissues were collected for analyses. Tumour length and width were measured by vernier caliper daily and tumour volume was calculated as length × width^2^/2.

### Statistical analysis

Results were expressed as mean±s.e.m. Statistical significance of differences between groups was analysed by Student’s *t*-test for two groups and analysis of variance for groups greater than two. Significance was accepted when *P*<0.05.

## Author contributions

Q.L., T.H., L.H., X.H., X.T., H.Z., L.H., W.P., L.Z., H.S., Y.Y., S.D., Q.X. and C.H. performed the research and analysed data. J.F., H.Z. and L.H. provided human samples. Q.L. and B.Z. designed the research and analysed data. T.Q. provided a mouse line. B.Z., J.W. and K.R. wrote the manuscript.

## Additional information

**How to cite this article**: Liu, Q. *et al*. Genetic targeting of sprouting angiogenesis using Apln-CreER. *Nat. Commun.* 6:6020 doi: 10.1038/ncomms7020 (2015).

## Supplementary Material

Supplementary Information Supplementary Figures 1-21 and Supplementary Table 1
