# Supplementary material for: Genetic targeting of sprouting angiogenesis using Apln-CreER
Source: Nat Commun. 2015 Jan 19;6:6020. doi: 10.1038/ncomms7020 (PMC4309445; doi:10.1038/ncomms7020)
Supplement: Supplementary Information — Supplementary Figures 1-21 and Supplementary Table 1 [file ncomms7020-s1.pdf]

Genetic Targeting of Sprouting Angiogenesis Using Apln-CreER

(NCOMMS-14-10623B)

Supplementary Information

Supplementary Figure 1 – 21

Supplementary Table 1

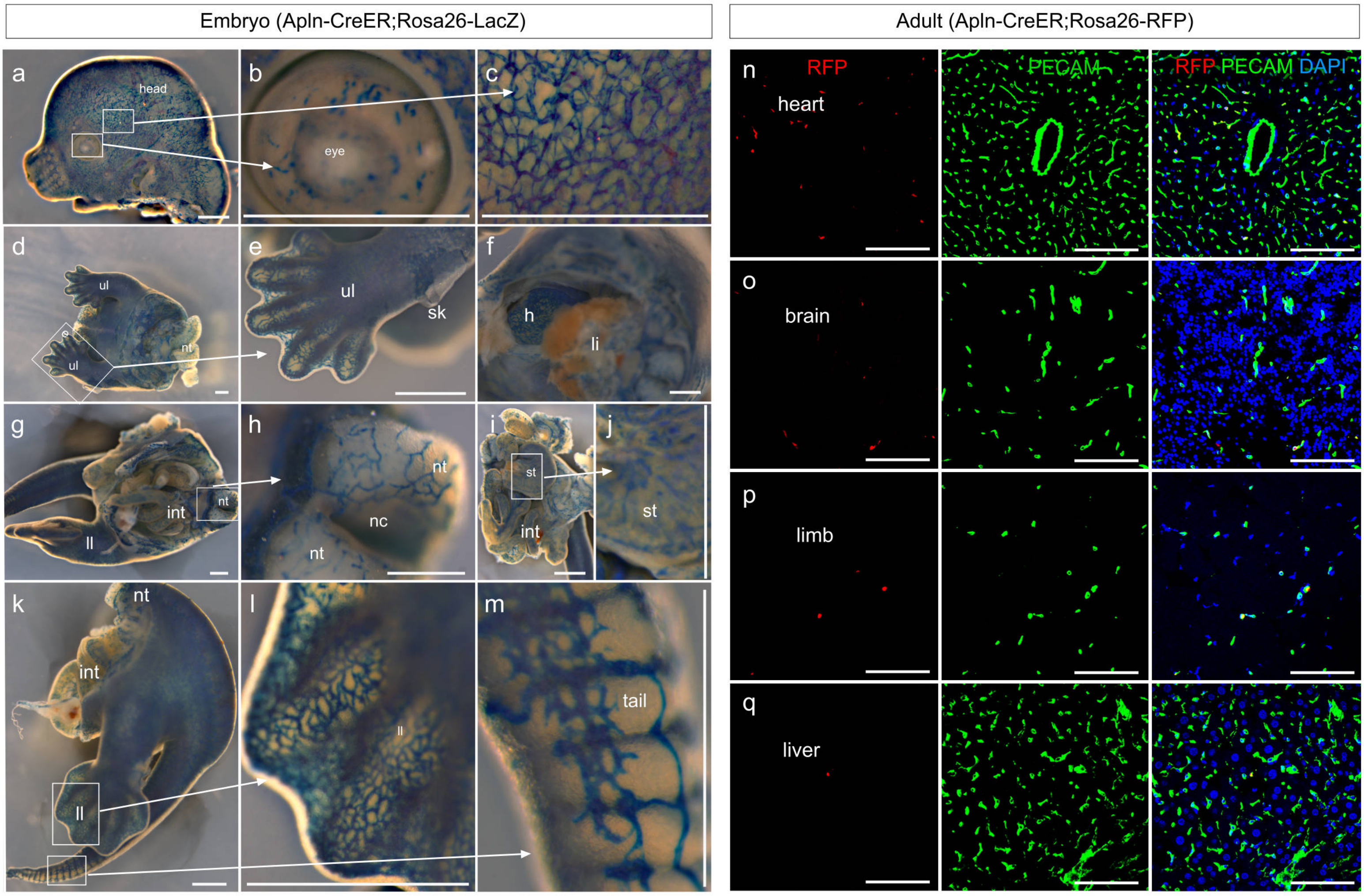

Supplementary Figure 1. Apln-CreER robustly label vascular endothelial cells in the developing embryo, but little activity in the adult. (a-m) Whole mount view of E13.5 Apln-CreER;Rosa26$^{LacZ/+}$ tissues after X-gal staining. Tamoxifen was induced at E10.5. ul, upper limb; nt, neural tube; nc, neural canal; sk, skin; h, heart; li, liver; ll, lower limb; st, stomach; int, intestine. **(n-q)** 11 weeks' old Apln-CreER;Rosa26$^{RFP/+}$ adult mice were treated with tamoxifen, and tissues were harvested 1 week later. Tissue sections were stained with RFP, PECAM and DAPI. Images were representative of 3 individual samples. Scale bars, 1 mm in **a-m**; 100 μm in **n-q**.

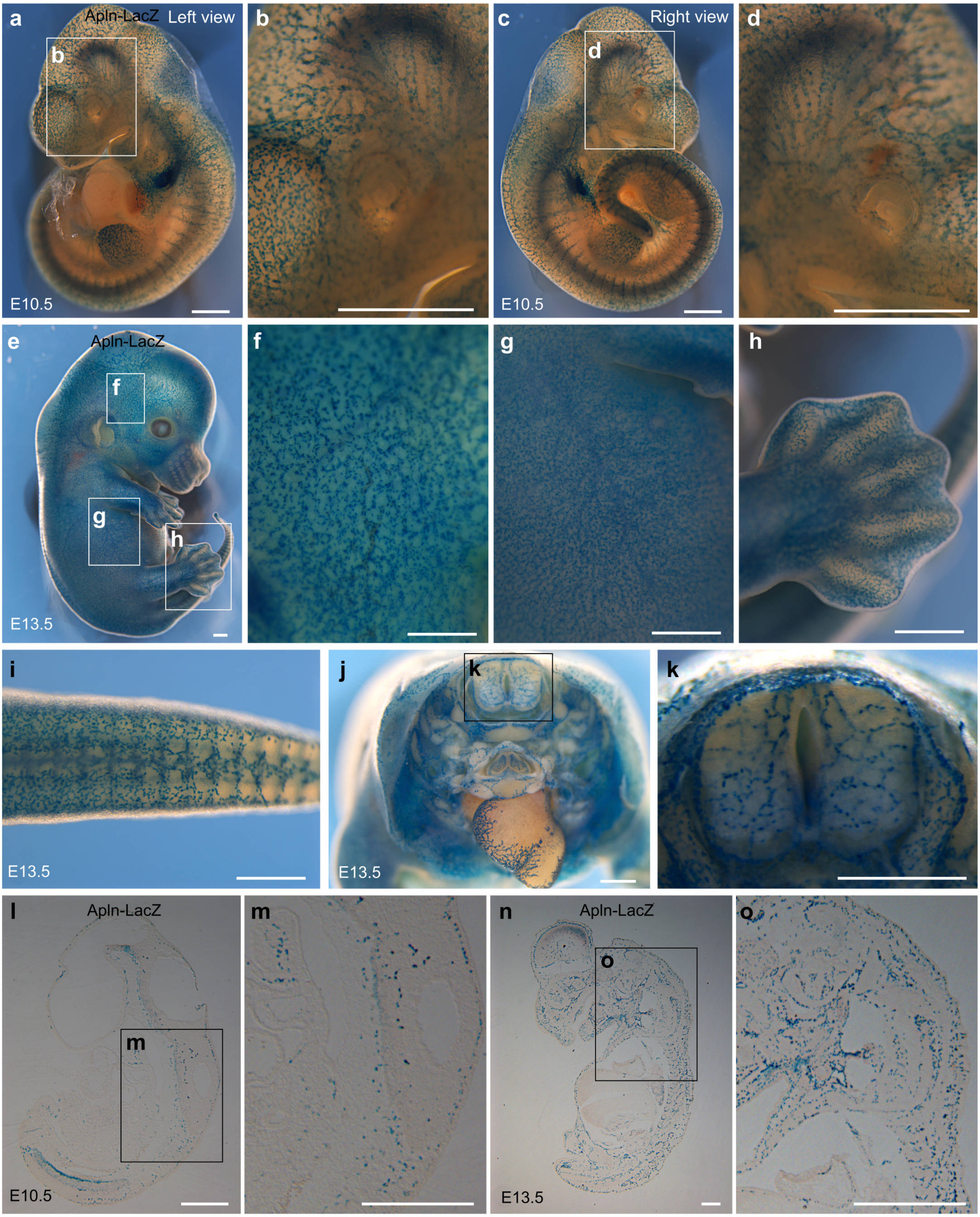

Supplementary Figure 2. Expression of APLN in E10.5 and E13.5 embryos. (a-d) Wholemount X-gal staining of E10.5 Apln-LacZ embryos. **(e-k)** Wholemount X-gal staining of E13.5 Apln-LacZ embryos. **(l-o)** X-gal staining of E10.5 and E13.5 Apln-LacZ embryonic sections. n = 4 to 5 embryos examined at each time point. Scale bars, 500 μm.

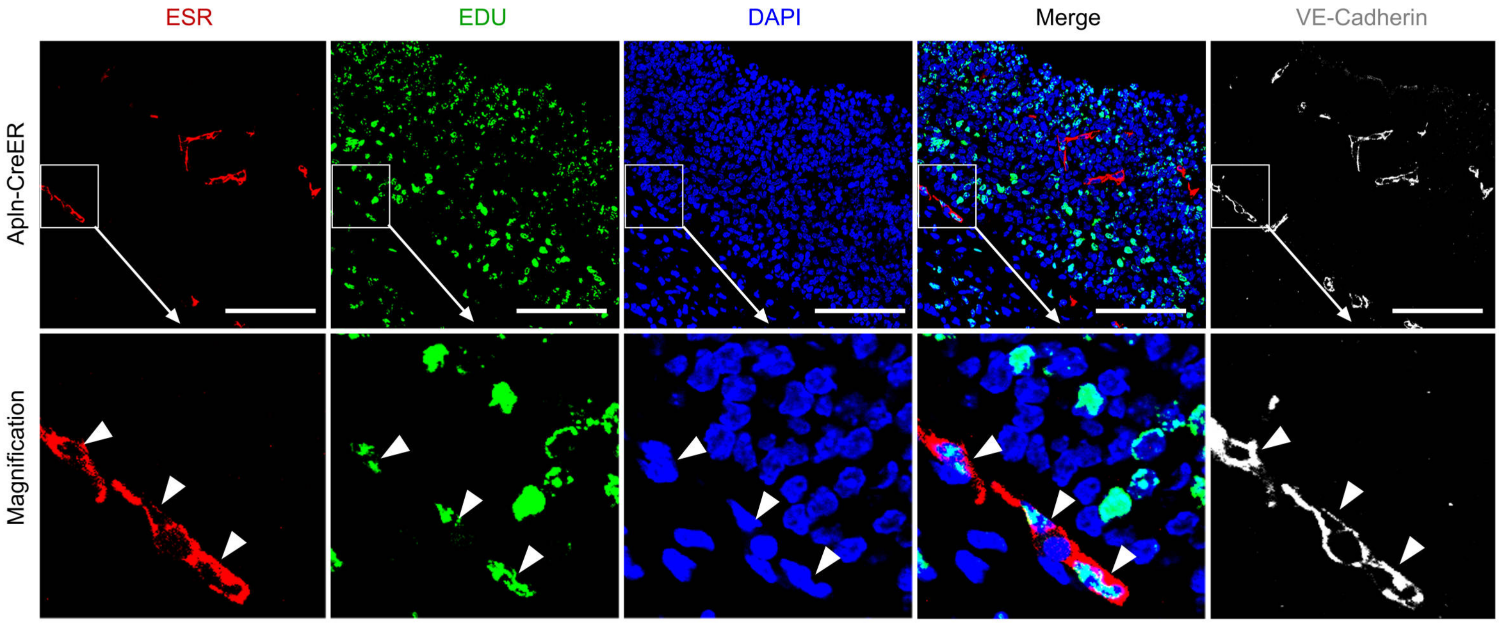

Supplementary Figure 3. APLN⁺ cells are proliferating endothelial cells in E10.5 embryos. Immunostaining of ESR, EDU, VE-Cadherin and DAPI on E10.5 Apln-CreER embryos. Representative of 3 individual samples. Scale bars, 100 μm.

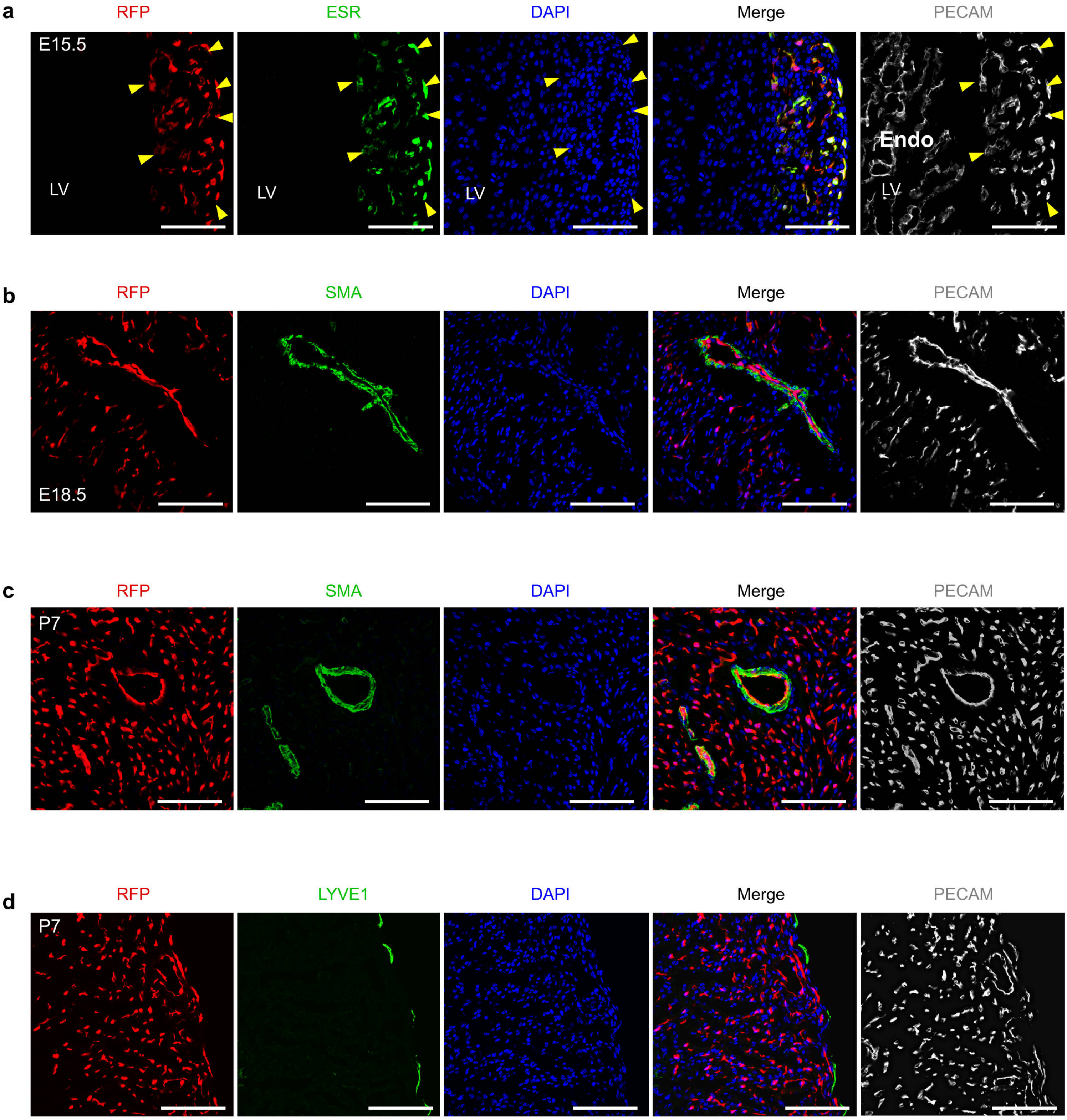

Supplementary Figure 4. Apln-CreER specifically label vascular endothelial cells. (a) Apln-CreER labels coronary vascular endothelial cells (yellow arrowheads), but not endocardial endothelial cells (Endo). **(b, c)** Apln-CreER does not label SMA^{+} vascular smooth muscle cells. **(d)** Apln-CreER does not label LYVE1^{+} lymphatic vascular endothelial cells. Tamoxifen was induced at E10.5. Each figure is representative of 3 individual samples. Scale bars, 100 μm.

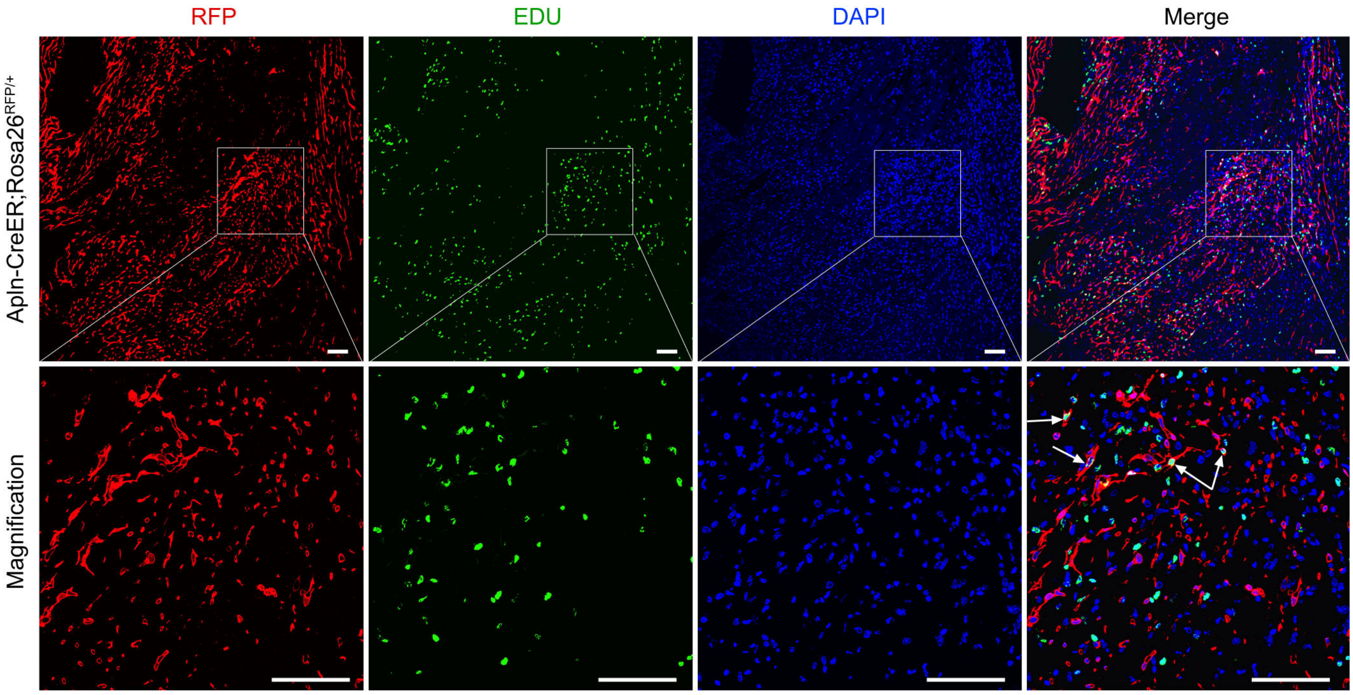

Supplementary Figure 5. Proliferation of Apln-CreER labeled vascular endothelial cells in MI region. Immunostaining of RFP, EDU and DAPI on heart sections 3 days after injury. 6.22% RFP^+ endothelial cells were EDU^+ in heart tissues. EDU was injected intraperitoneally 24 hours before heart retrieval. Each image is representative of 3 individual samples. Scale bar, 200 μm.

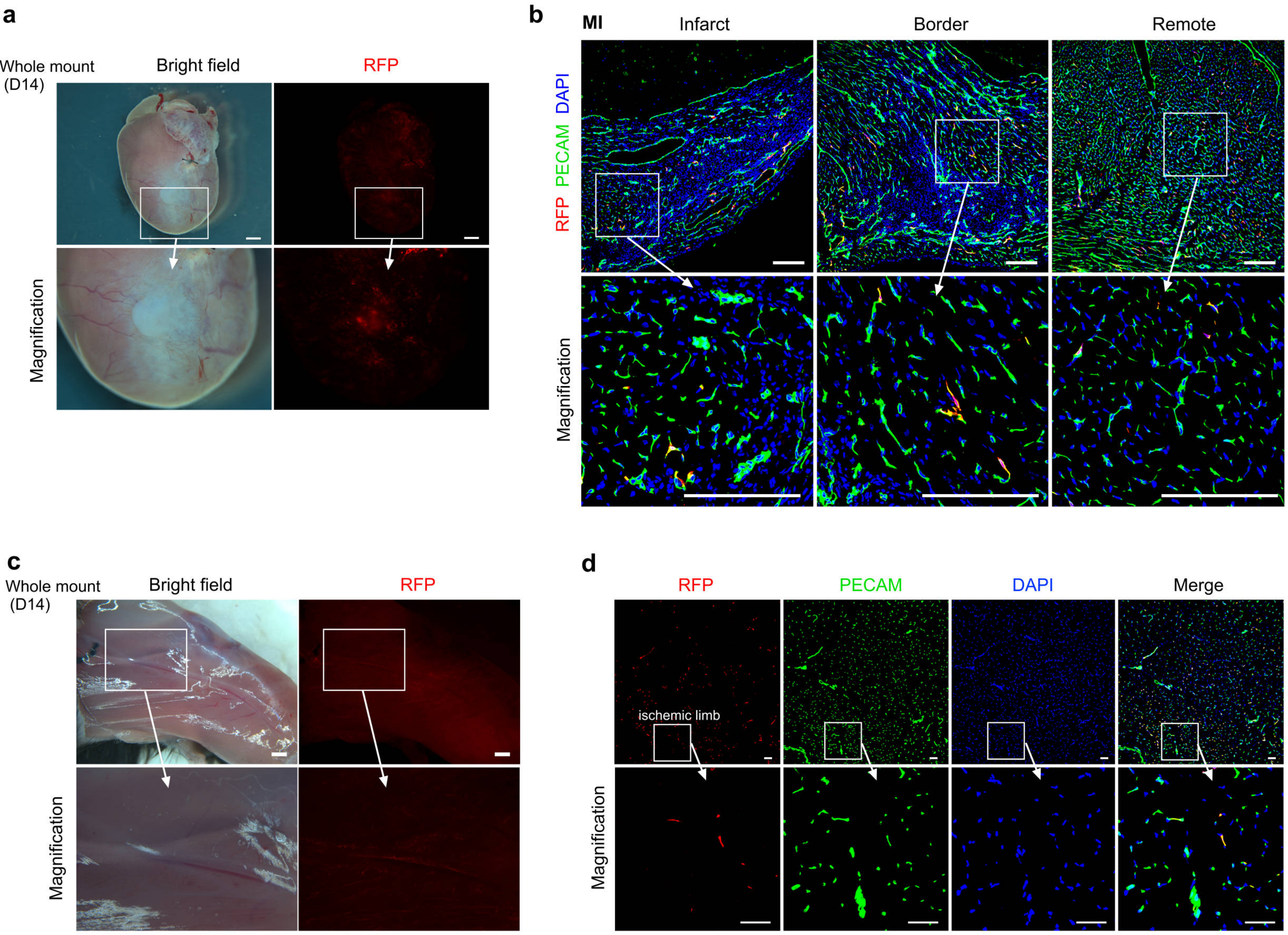

Supplementary Figure 6. Leakiness of Apln-CreER in endothelial cells labeling in heart and limb. (a) Whole mount view of Apln-CreER;Rosa26$^{RFP/+}$ heart 14 days after MI. No tamoxifen was treated. **(b)** Immunostaining of RFP, PECAM and DAPI on Apln-CreER;Rosa26$^{RFP/+}$ heart 14 days after MI. No tamoxifen was treated. Image is representative of four individual hearts. **(c)** Whole mount view of the hindlimb at 14 days post hindlimb ischemia. Apln-CreER;Rosa26$^{RFP/+}$ mice did not receive tamoxifen after injury. **(d)** Immunostaining of the lineage marker RFP, PECAM and DAPI in the injured hindlimb in the absence of tamoxifen treatment. White boxed areas are magnified in the panels below. Scale bars, 1 mm in **a**,**c**; 100 µm in **b**,**d**.

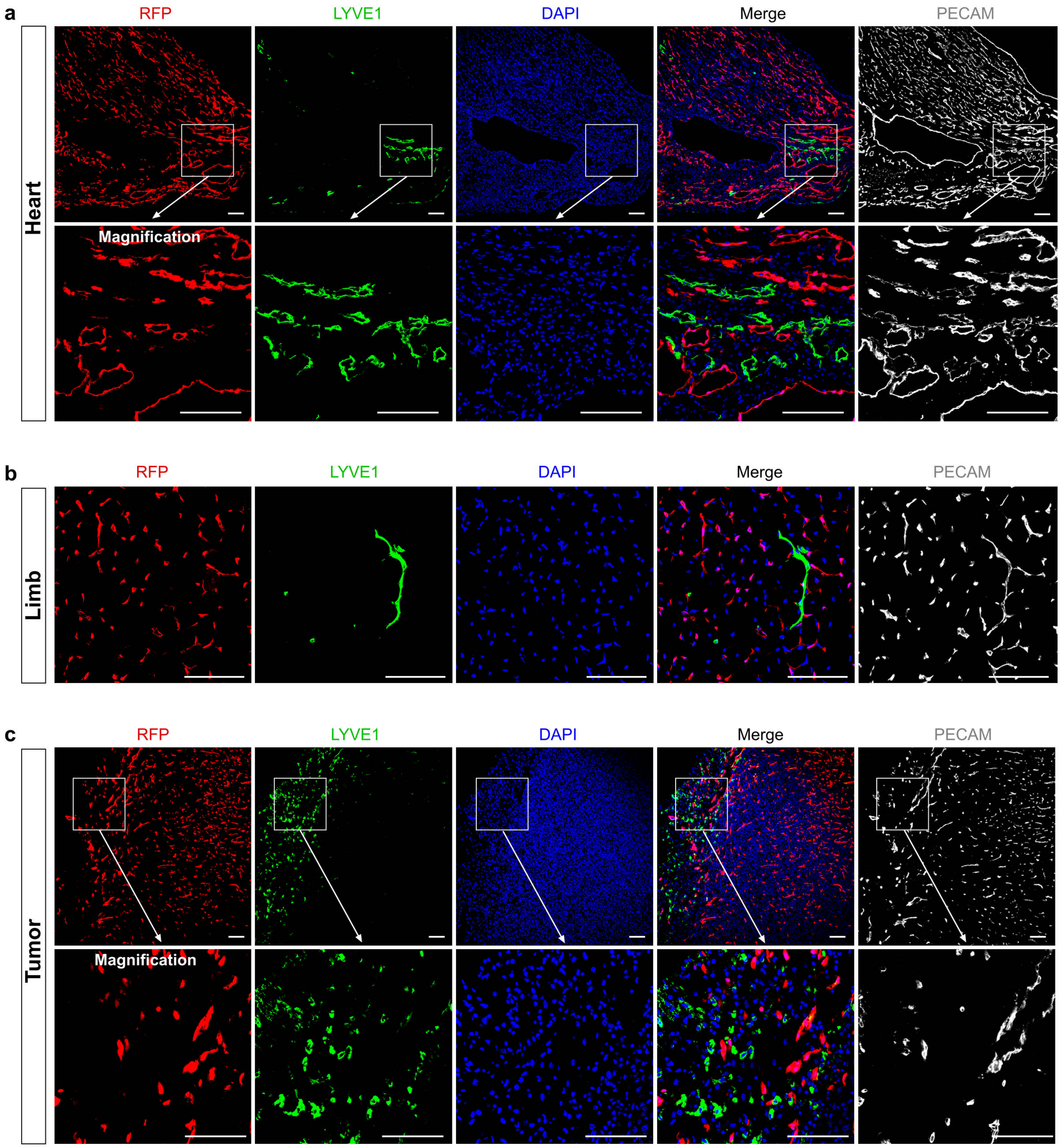

Supplementary Figure 7. Apln-CreER labeled cells do not adopt lymphatic vascular endothelial cell fate. (a-c) Immunostaining of genetic marker RFP, lymphatic vessel marker LYVE1 and PECAM on ischemic heart, hindlimb tissues and tumor samples. Apln-CreER does not label LYVE1^{+} lymphatic vascular endothelial cells in injured heart, limb or tumors. Each image was representative of 3 individual samples. Scale bars, 100 µm.

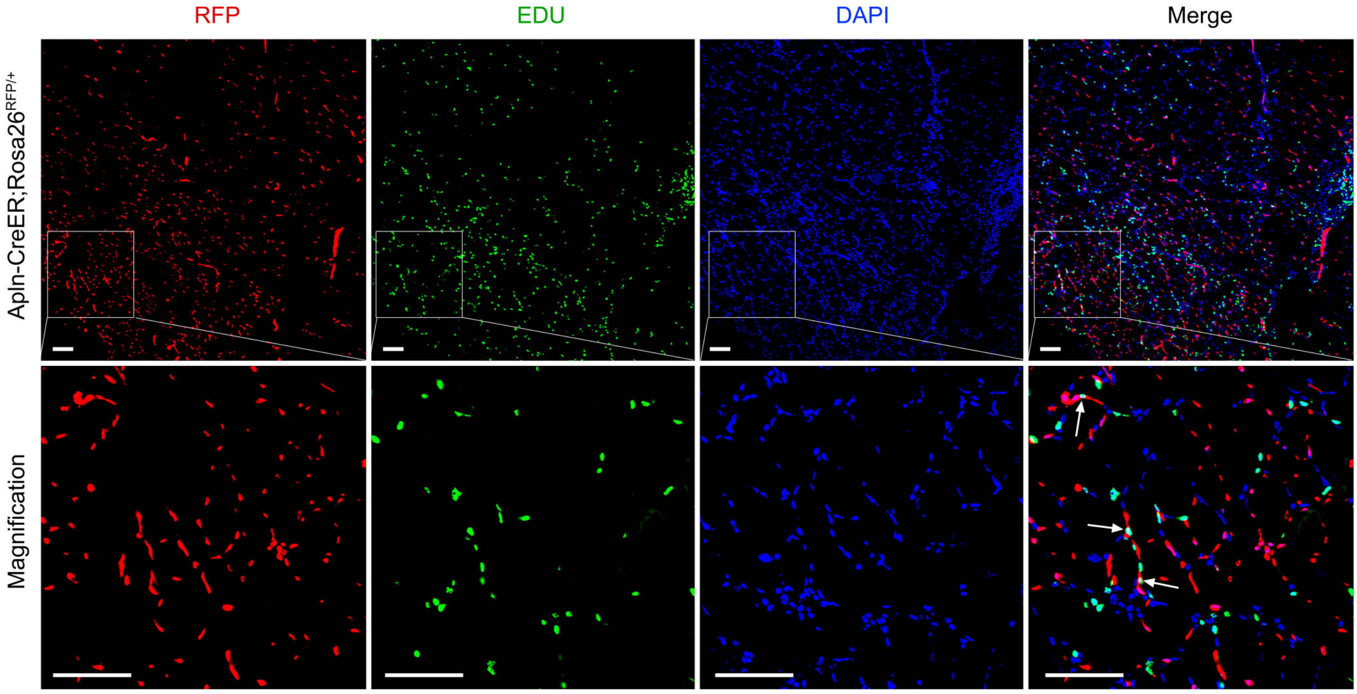

Supplementary Figure 8. Proliferation of Apln-CreER labeled vascular endothelial cells in injured hindimb. Immunostaining of RFP, EDU and DAPI on hindlimb sections 3 days after injury. 3.85% RFP^+ endothelial cells were EDU^+ in hindlimb tissues. EDU was injected intraperitoneally 24 hours before limb retrieval. Representative of 3 individual samples. Scale bars, 200 μm.

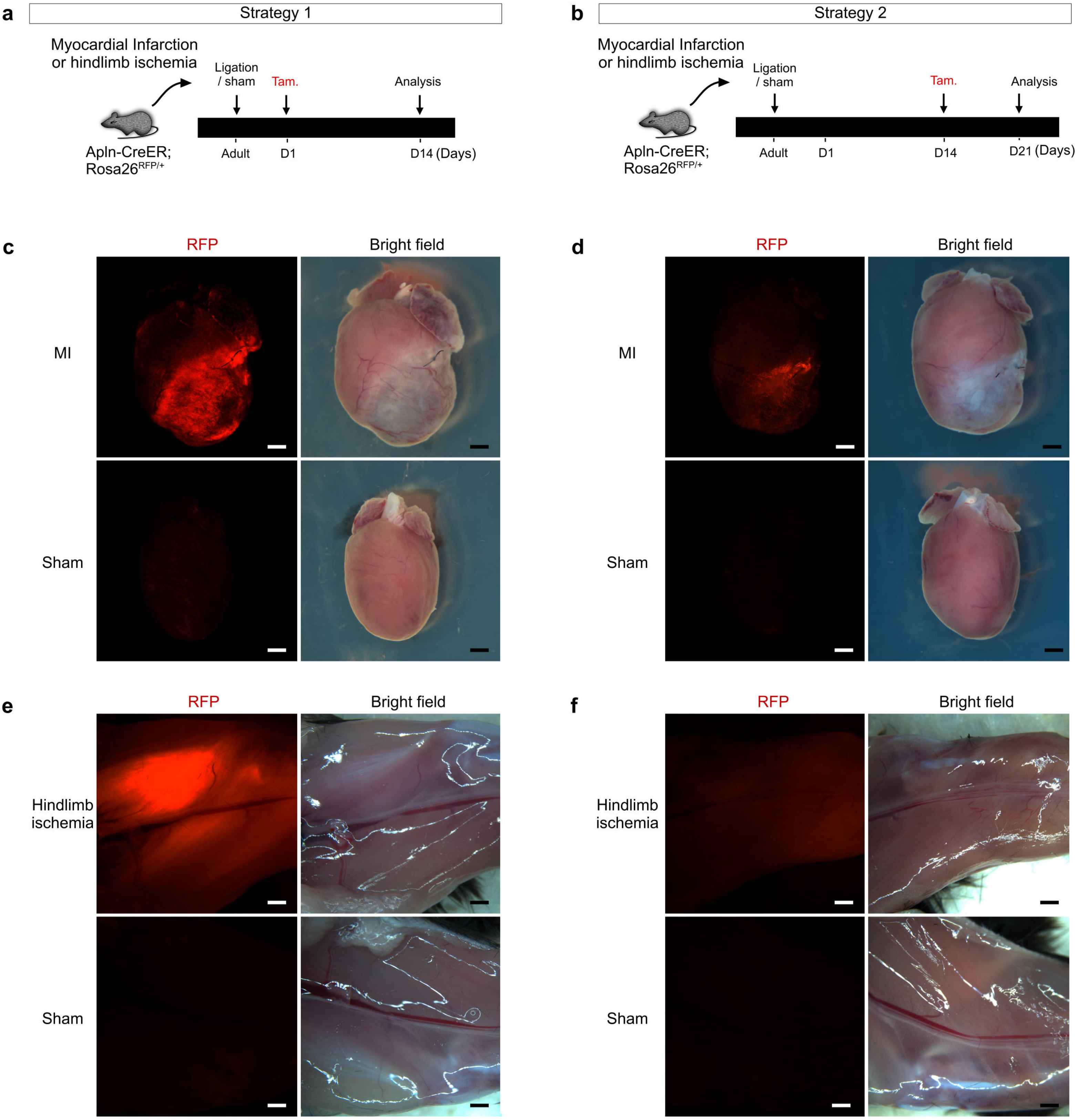

Supplementary Figure 9. Lower labeling of vessels by tamoxifen treatment at 2 weeks after injury. (a, b) Schematic figures showing two different strategies of tamoxifen induction. **(c, d)** Wholemount fluorescence or bright field pictures of hearts after MI or sham treatment. Compared with MI heart in strategy 1, the MI heart in strategy 2 have reduced RFP singal, indicating lower labeling of vessels when tamoxifen was administered at two weeks after MI. **(e, f)** Wholemount fluorescence or bright field pictures of hindlimbs after ligation or sham treatment. Compared with injured hindlimb in strategy 1, the injured hindlimb in strategy 2 have reduced RFP signal, indicating lower labeling of vessels when tamoxifen was administered at two weeks after ligation. Representative of at least 3 samples per group. Scale bars, 1 mm.

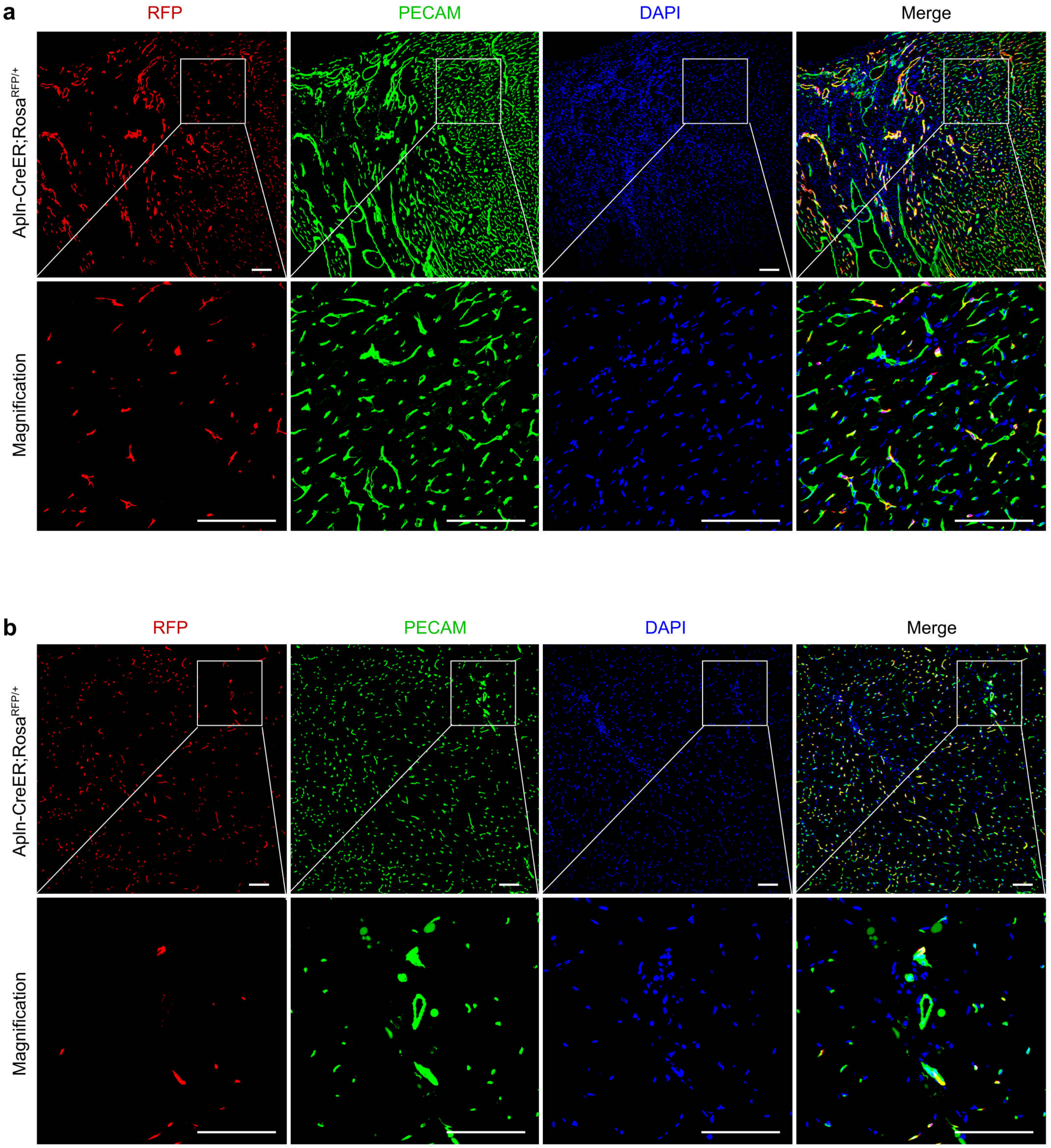

Supplementary Figure 10. Apln-CreER labeled vascular endothelial cells. (a-b) Immunostaining of RFP, PECAM and DAPI on Apln-CreER;Rosa26$^{RFP/+}$ mice at three weeks after MI (a) or hindlimb ischemia (b). Tamoxifen was induced at two weeks after MI or hindlimb ischemia. Representative of 3 individual samples. Scale bars, 100 µm.

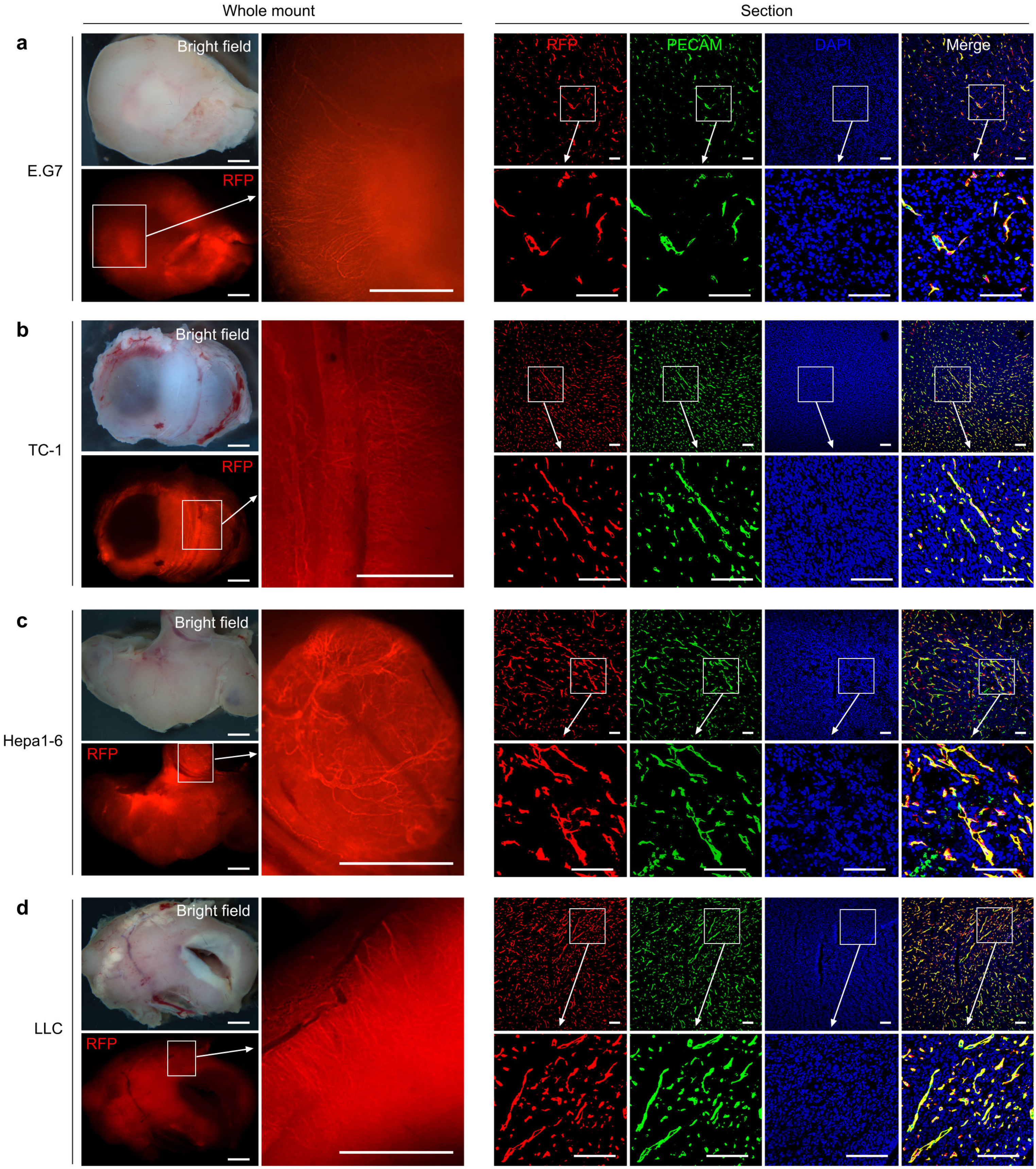

Supplementary Figure 11. Apln-CreER efficiently labels tumor-induced vascular ECs. (a-d) Whole mount phase contrast, whole mount live fluorescence, and indirect immunofluorescent staining for RFP and PECAM on sectioned tissue (left to right, respectively) showed Apln-CreER strongly lables tumor ECs. Tumors were generated by subcutaneous implant of oncogenic cell lines in Apln-CreER;Rosa26$^{RFP/+}$ adult mice. Tamoxifen was administered 1 day following implant. Four different tumor cells lines (E.G7, TC-1, Hepa1-6 and LLC) were tested. Each image is representative of at least four individual samples. Scale bars, 1 mm in **a-d** whole mount images; 100 μm in **a-d** section images.

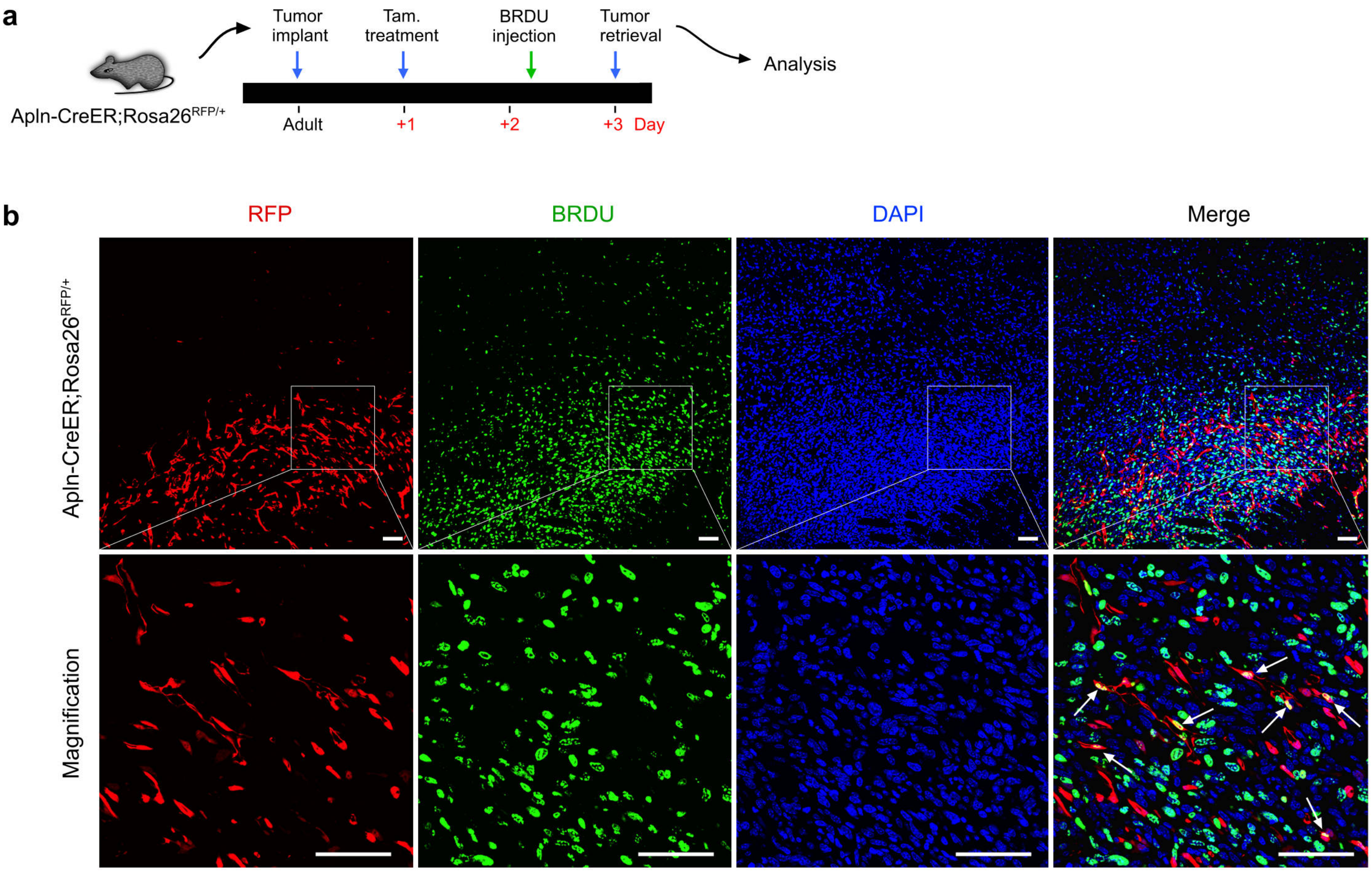

Supplementary Figure 12. Proliferation of Apln-CreER labeled vessels in tumor. (a) Strategy of tumor implantation and tamoxifen treatment in Apln-CreER;Rosa26$^{RFP/+}$ mice. **(b)** Immunostaining of RFP, BRDU and DAPI on tumor samples showed 19.56% proliferating endothelial cells (BRDU^{+}) among RFP^{+} cells (white arrows). n = 3. Scale bars, 200 µm.

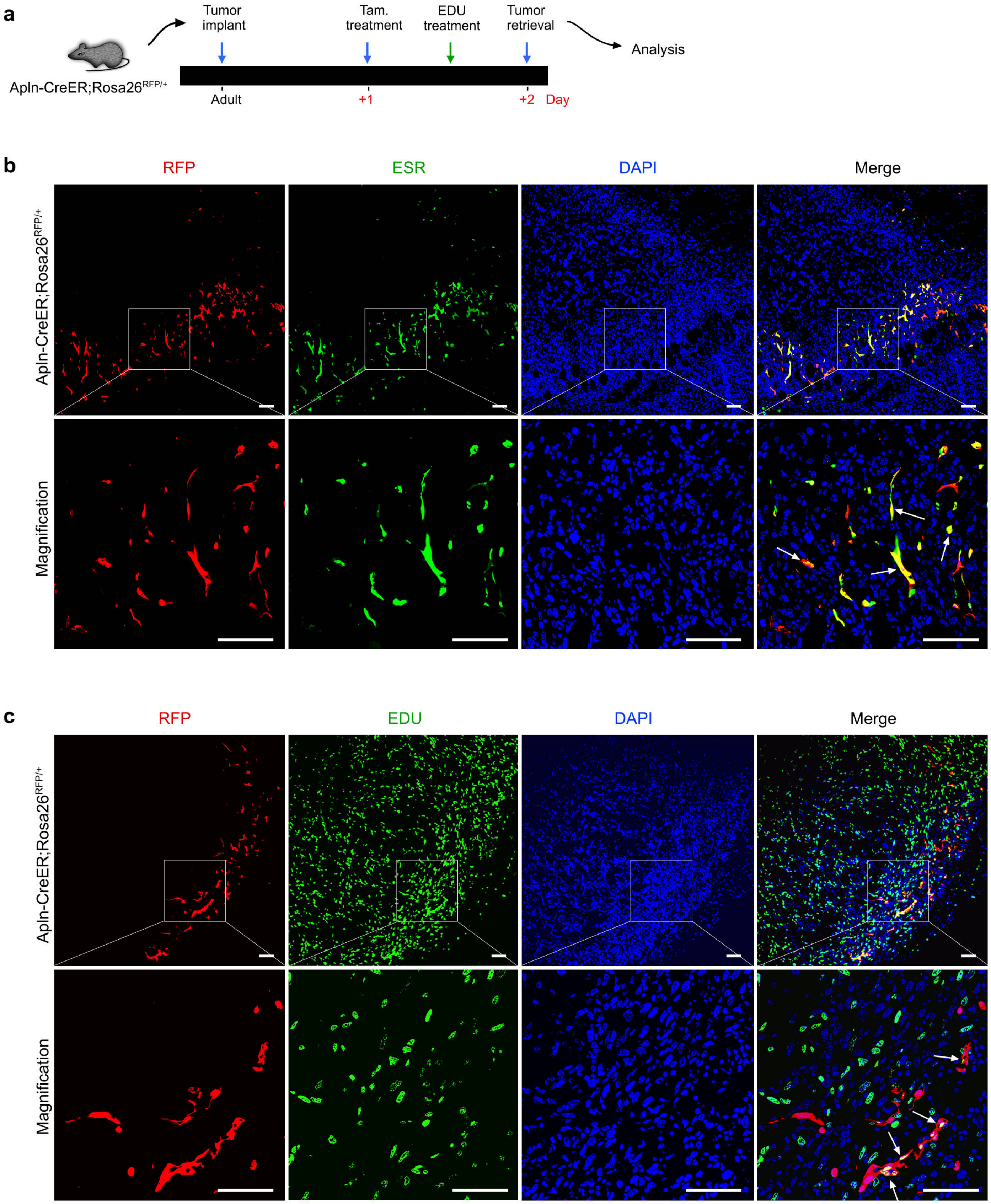

Supplementary Figure 13. Apln-CreER labeled preliminary vessels in tumor. (a) Strategy of tumor implantation, tamoxifen induction and EDU treatment in Apln-CreER;Rosa26$^{RFP/+}$ mice. **(b)** RFP, ESR and DAPI staining of tumor samples retrieved two days after implantation. Most Apln-derived cells (RFP^{+}) were expressing APLN (detected by ESR, white arrows). **(c)** Immunostaining of RFP, EDU and DAPI on tumor samples showed 11.48% proliferating endothelial cells (EDU^{+}) among RFP^{+} cells (white arrows). n = 3. Scale bars, 200 μm.

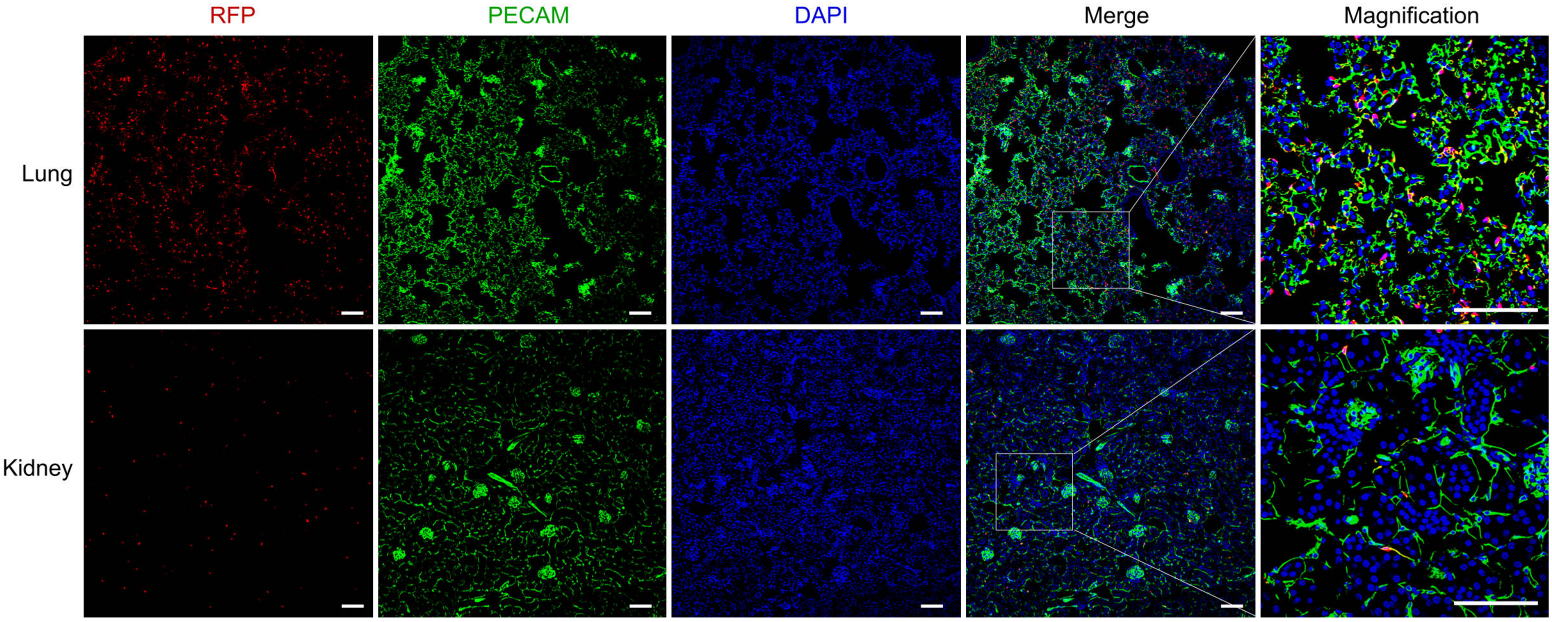

Supplementary Figure 14. Apln-CreER lineage tracing in lung and kidney. Immunostaining of lineage marker RFP, vascular endothelial cell marker PECAM and nuclei marker DAPI on Apln-CreER;Rosa26$^{RFP/+}$ tissue sections. Tamoxifen was treated about 10 days before analysis. Apln-CreER labels 30.11% and 4.75% vascular endothelial cells in lung and kidney respectively. Scale bars, 100 μm.

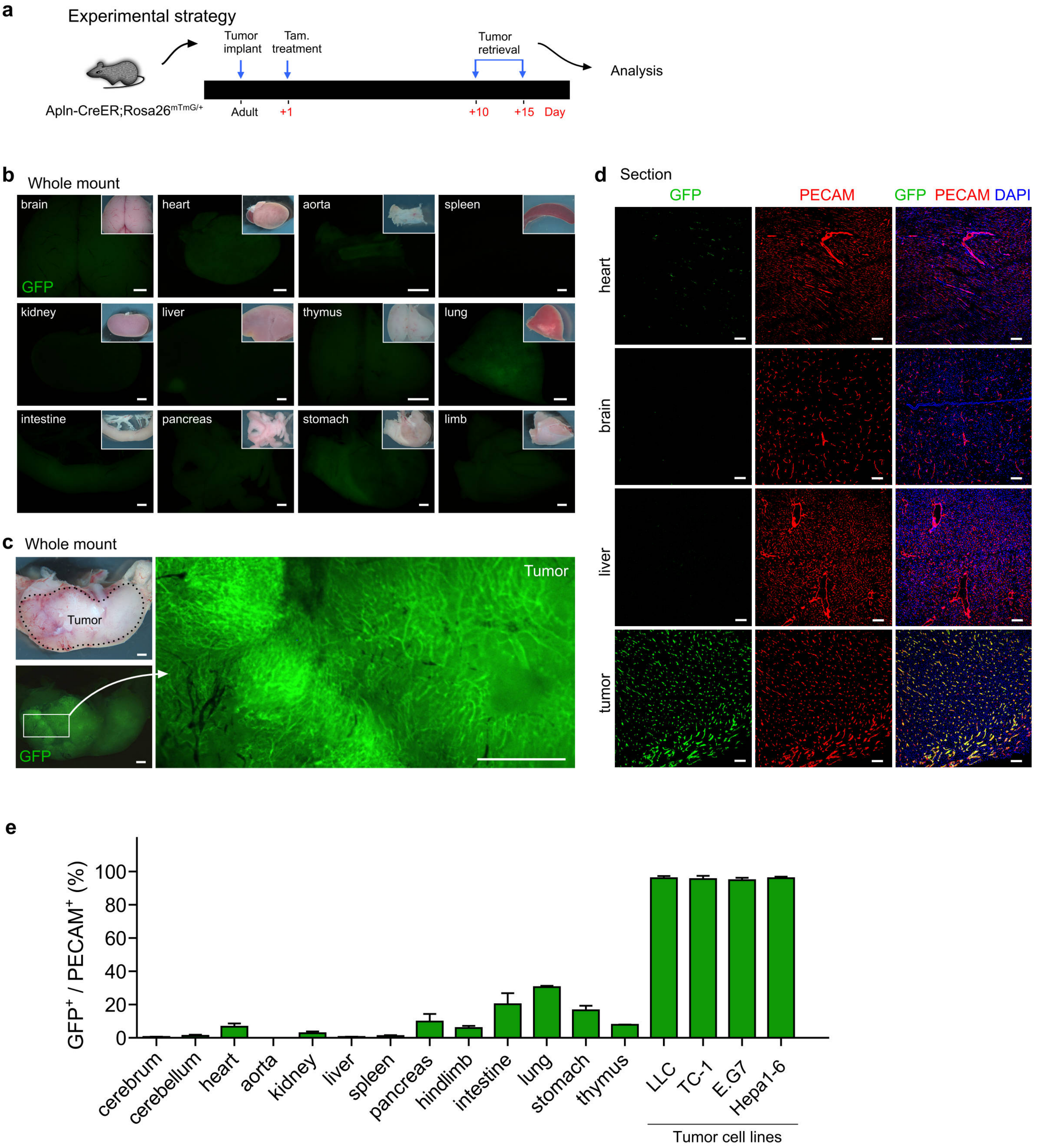

Supplementary Figure 15. Apln-CreER labels tumor angiogenic vessels. (a) Schematic model of the time course of tumor implantation, tamoxifen-induced genetic labeling, and tumor collection. **(b,c)** Whole mount view of various organs and a tumor from an Apln-CreER;Rosa26$^{mTmG/+}$ mouse. **(d)** Immunostaning for GFP, PECAM and DAPI of heart, brain, liver and tumor tissue. Images are representative of 3 - 4 individual samples. **(e)** Quantification of the percentage of GFP^{+} vascular endothelial cells in various organs and tumors. Values are shown as means ± s.e.m.; n = 3 - 4 for each sample. Scale bars, 1 mm in **b,c**; 100 μm in **d**.

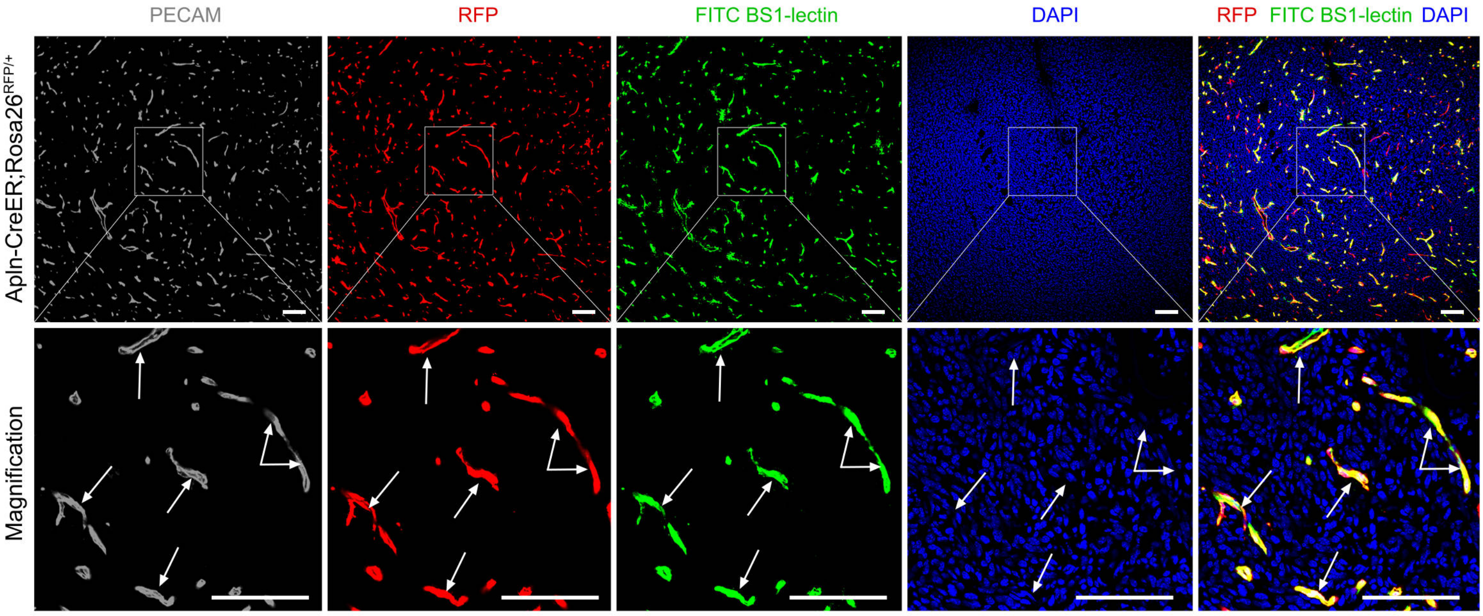

Supplementary Figure 16. Apln-CreER labeled tumor vessels are functional. Immunostaining of PECAM, RFP, FITC BS1-lectin and DAPI on tumor samples from Apln-CreER;Rosa26$^{RFP/+}$ mice. BS1-lectin^{+}RFP^{+} tumor vessels (white arrows) indicate there is blood flow in these vessels. FITC labeled BS1-lectin was injected into mice inferior caval vein 1 hour before tumor retrieval. Representative of 3 individual samples. Scale bars, 100 μm.

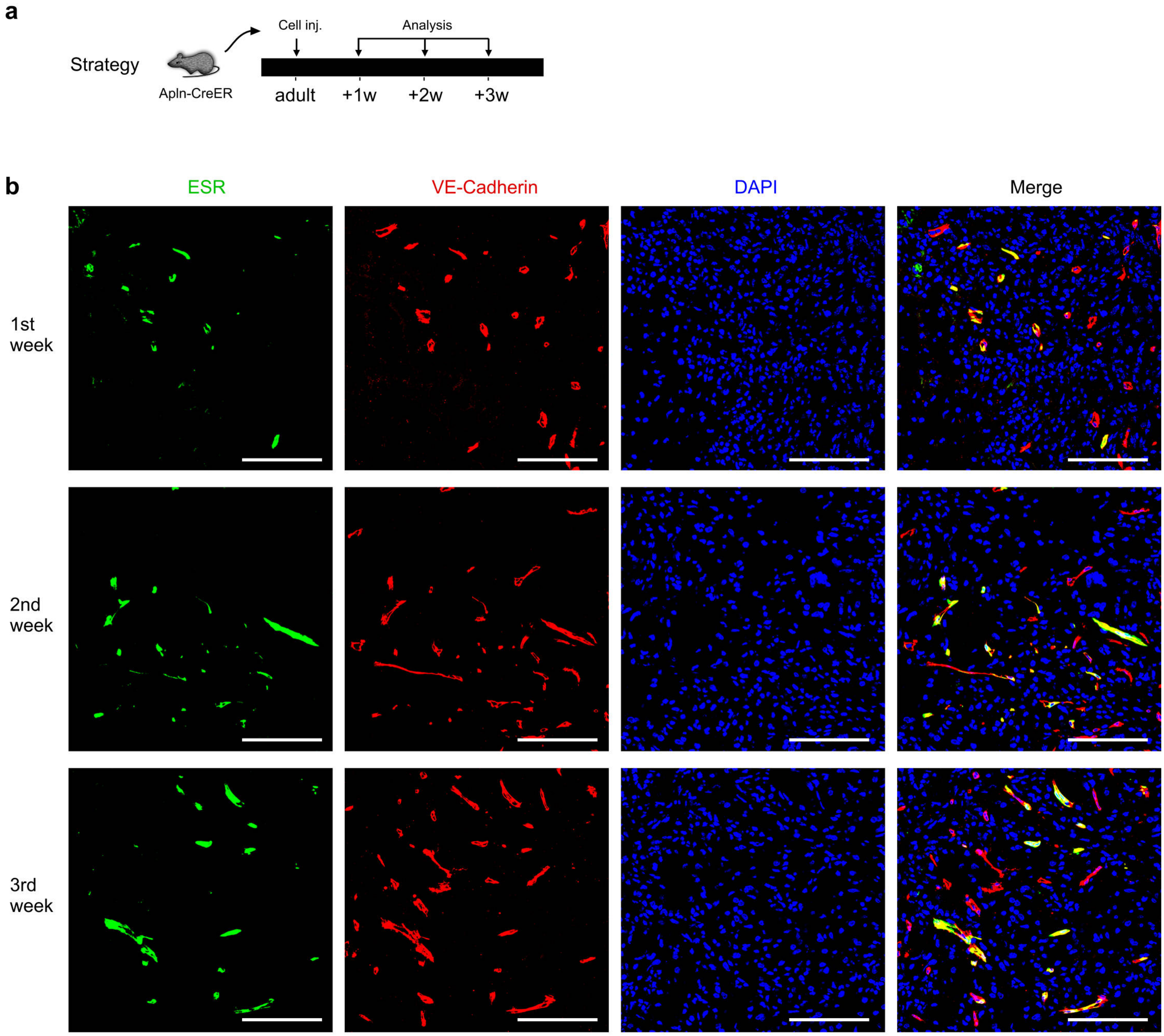

Supplementary Figure 17. Expression of APLN in tumor endothelial cells. (a) Strategy of orthotopic liver tumor model establishment in Apln-CreER mice. Liver samples were collected for analysis at 1, 2, 3 weeks after tumor implantation. **(b)** Immunostaining of estrogen receptor (ESR) as surrogate for APLN expression, endothelial cell marker VE-Cadherin and nuclei dye DAPI on liver tumor sections collected at different time after implantation. Representative of 3 samples for each time point. Scale bars, 100 μm.

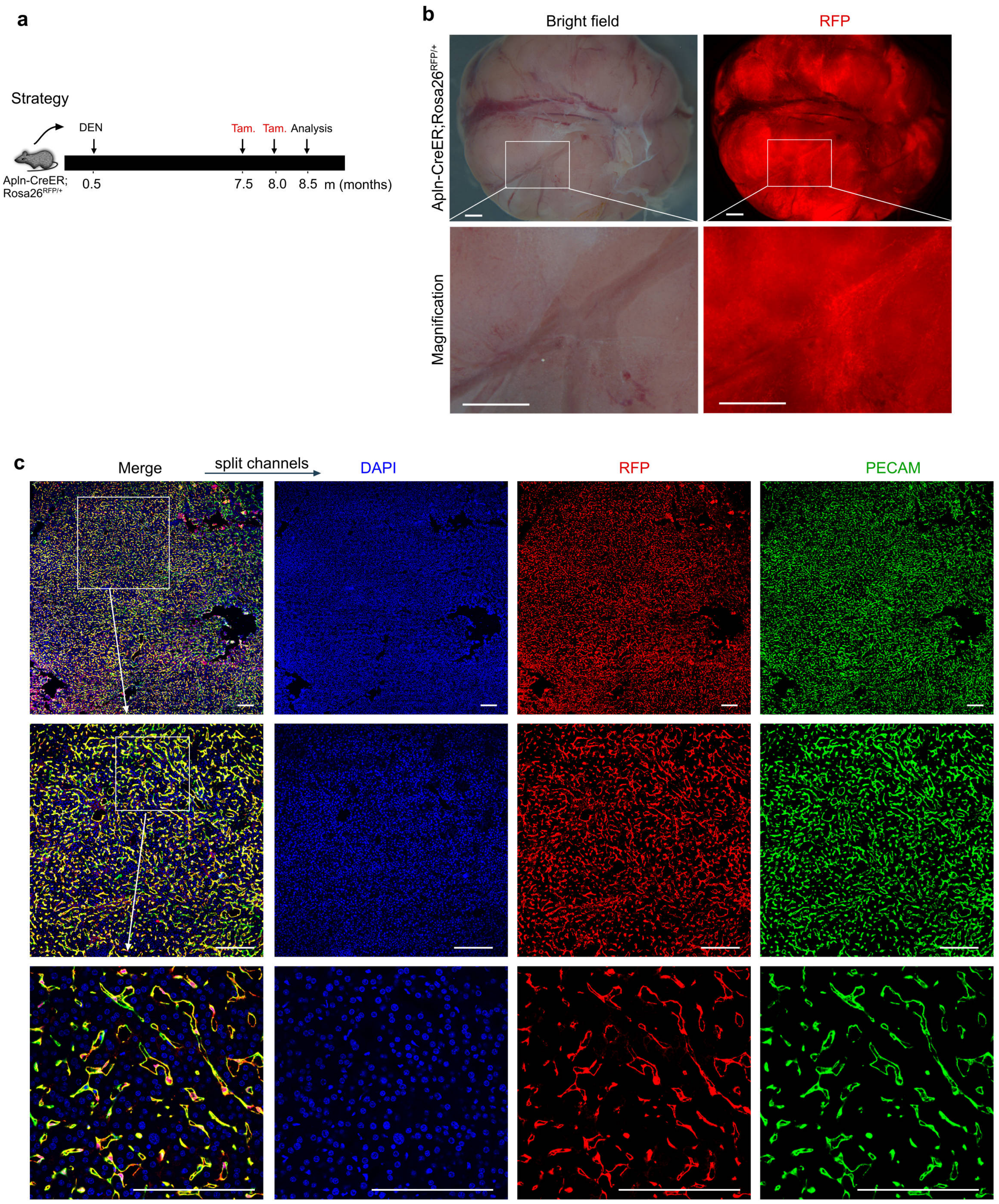

Supplementary Figure 18. Apln-CreER labels tumor neovasculature in DEN-induced mesentery tumors. (a) Scheme for DEN-mediated tumor induction and Apln-lineage tracing of the tumor endothelium. **(b)** Phase contrast and live fluorescence whole mount view of mesentery tumors (left to right) and magnified views below. **(c)** Indirect immunofluorescence performed on DEN-induced mesentery tumor tissue for DAPI, RFP and PECAM shows that the Apln-CreER lineage contributes extensively to the mesentery tumor endothelium. Views (top to bottom) are successive magnifications. Scale bars, 1 mm in **b**; 200 μm in **c**.

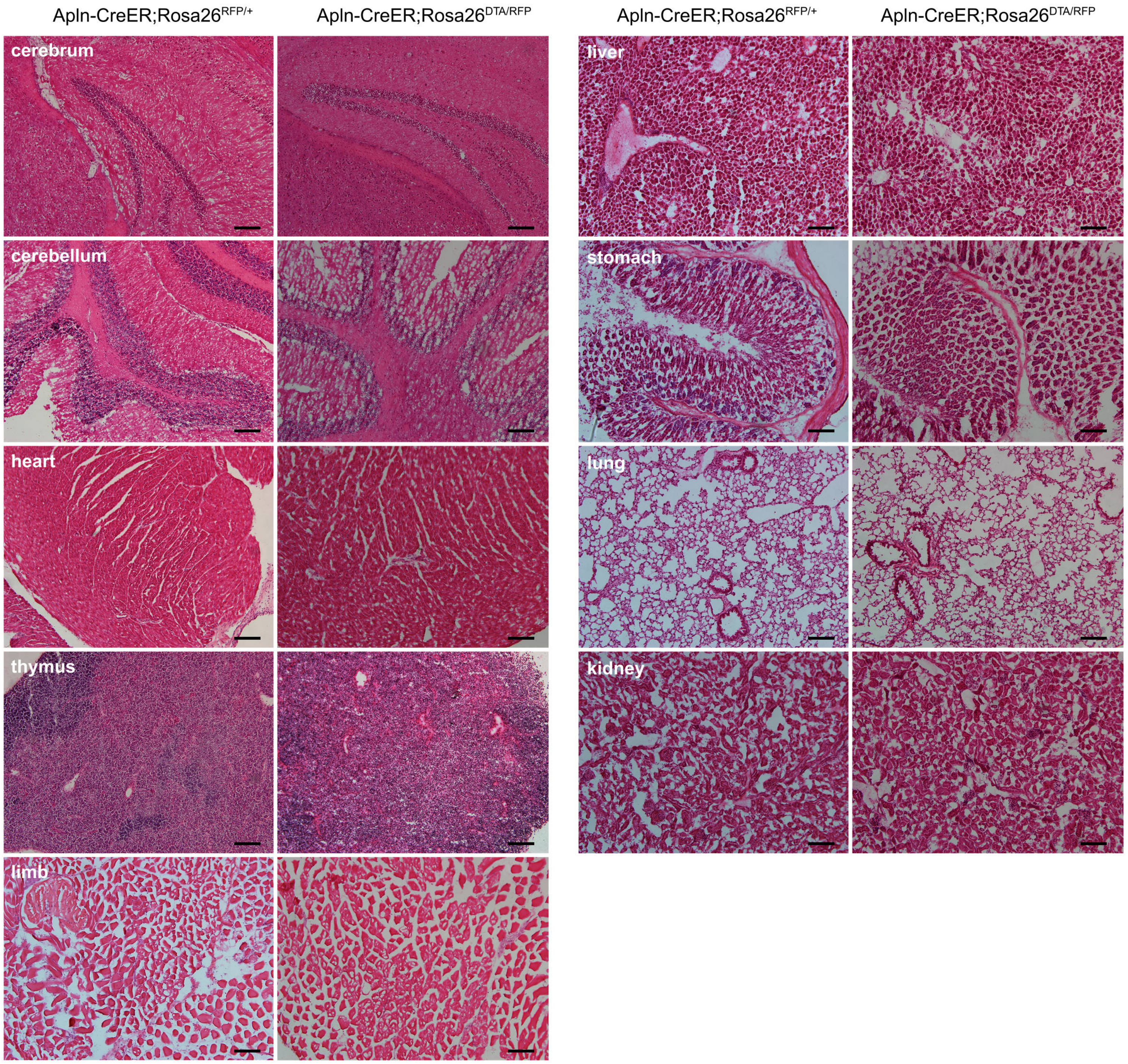

Supplementary Figure 19. Normal gross morphology in organs following Apln-CreER/DTA-mediated tumor vasculature ablation. H&E staining of tissue from Apln-CreER;Rosa26$^{RFP/+}$ and Apln-CreER;Rosa26$^{DTA/RFP}$ mice, both treated with tamoxifen. Images are representative of 3 individual samples per group. Scale bars, 200 μm.

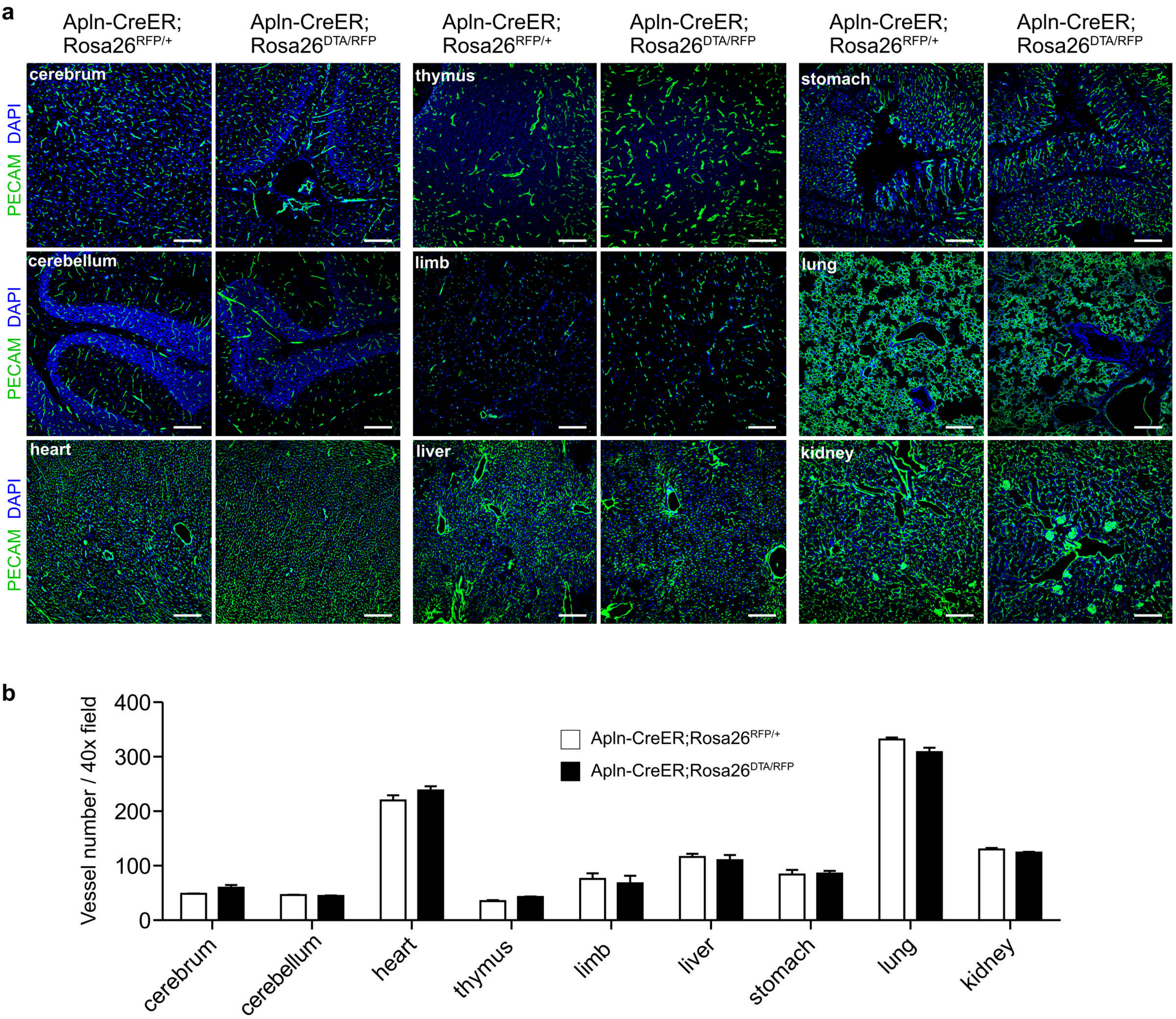

Supplementary Figure 20. Unaltered vascular density in non-tumorigenic tissues following Apln-CreER/DTA-mediated EC ablation. (a) PECAM and DAPI staining on non-cancerious tissue sections is indistinguishable in Apln-CreER;Rosa26$^{RFP/+}$ and Apln-CreER;Rosa26$^{DTA/RFP}$ mice following tamoxifen administration. **(b)** Quantification of vessel number per 40X field of view for various tissues (3 independent samples for each tissue examined). Values are shown as means ± s.e.m.; Scale bars, 200 μm.

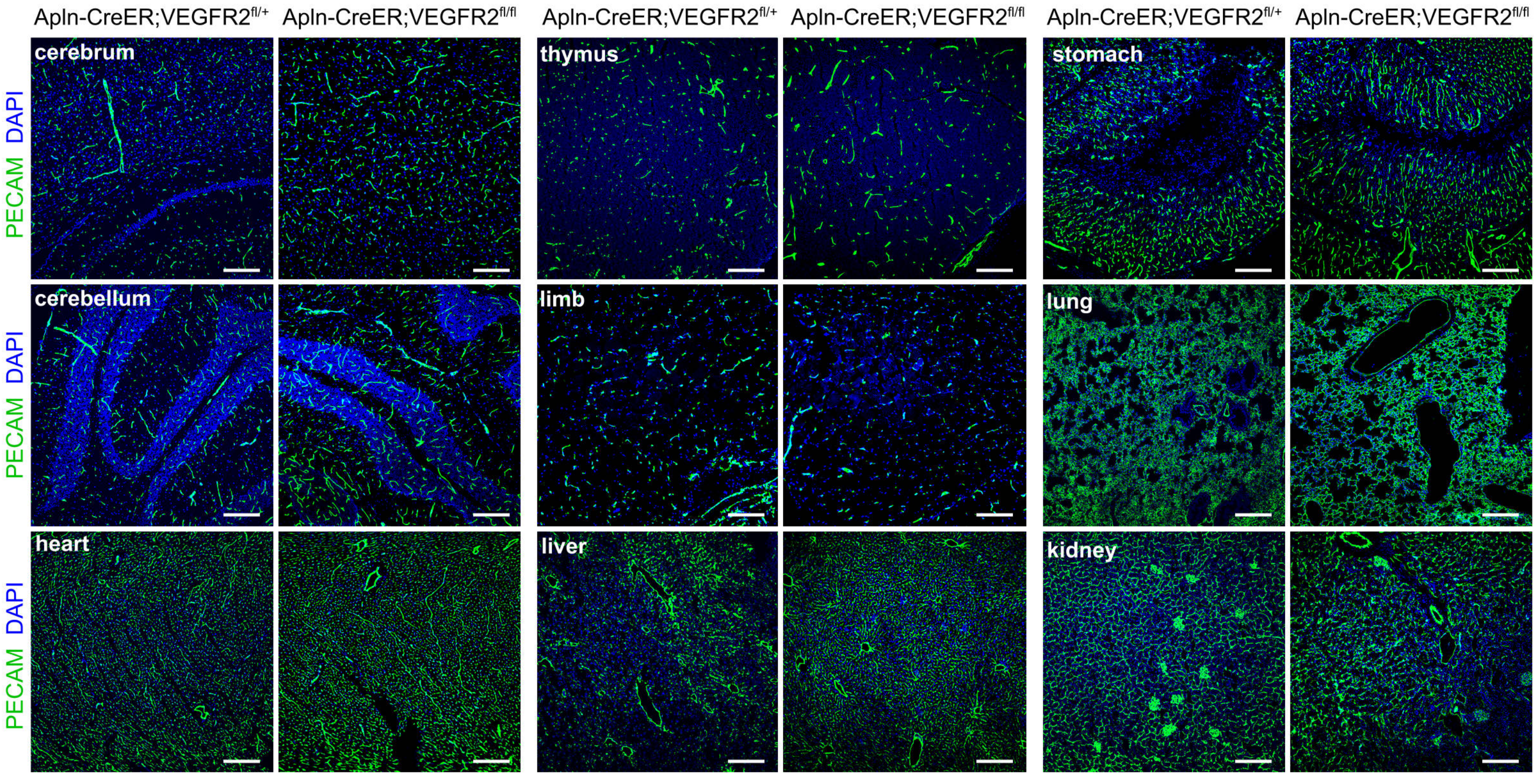

Supplementary Figure 21. Unaltered vascular density in non-tumorigenic tissues following Apln-CreER-mediated VEGFR2 deletion. PECAM and DAPI staining on non-cancerious tissue sections is indistinguishable in Apln-CreER;VEGFR2$^{fl/+}$ and Apln-CreER;VEGFR2$^{fl/fl}$ mice following tamoxifen administration. n = 3. Scale bars, 200 μm.

Supplementary Table 1. Human tumor sample information

Human hepatocellular carcinoma information

No.	age	sex	differential stage	TNM stage	size (cmxcm)
1	35	male	I	III	1.2×1
2	51	male	II	III	9.5×8
3	37	female	I	III	3.5×2
4	56	female	I	III	2.2×1.2
5	73	male	IIIB	III	14×10.8×9
6	59	male	I	III	2.6×2.2
7	70	male	II	III	12.8×7.2
8	57	female	II	III	6×5×3.8
9	56	female	I	II	1.6×1.4
10	49	male	IIIB	III	3×3
11	46	male	I	II	2×2
12	38	male	I	III	3.5×2.5
13	42	male	II	II-III	1.8×1.6、1.8×1.6
14	56	male	I	III	2×1.5
15	59	female	I	III	2.5×2

Human colon tumor samples

No.	age	sex	region	TNM phase			Stage
1	73	female	R	T3	N0	M0	II
2	73	male	CL	T2	N0	M1	IV
3	59	male	R	T4	N2	M0	III
4	75	male	R	T4	N0	M0	II
5	75	male	CR	T4	N1	M0	III
6	65	male	R	T4	N2	M0	III
7	65	male	CL	T4	N1	M1	IV
8	65	male	R	T4	N2	M0	III
9	37	female	CL	T4	N2	M0	III
10	72	male	R	T4	N0	M0	II
11	80	male	R	T3	N0	M0	II
12	75	male	R	T4	N0	M0	II

R	rectum
CL	colon(left)
CR	colon(right)
